# Wealth and mortality among late-middle-aged individuals in Norway: a nationwide register-based retrospective study

**DOI:** 10.1016/j.lanepe.2024.101113

**Published:** 2024-11-08

**Authors:** Alexi Gugushvili, Øyvind Nicolay Wiborg

**Affiliations:** Department of Sociology and Human Geography, University of Oslo, Moltke Moes vei 31, Harriet Holters Hus, N-0851, Oslo, Norway

**Keywords:** Wealth, Mortality, Inequality, Norway

## Abstract

**Background:**

In recent decades, we have observed rising wealth inequality while the pace of growth of life expectancy has slowed in many Western welfare democracies. There is scarce evidence, however, on links between wealth and mortality. The main methodological limitation in this area of scholarship is its inability to account for individuals' unobserved heterogeneity, such as personality and genetic factors, which could potentially affect both their wealth level and survival probabilities. This study aims to explore how wealth is linked to mortality risk in late-middle age, providing insights into the broader implications of socioeconomic status on health outcomes.

**Methods:**

In this study, we use high-quality register data on wealth and mortality for the entire population of Norway, one of the world's most advanced welfare states with a low income inequality level but a highly uneven distribution of wealth. We address some of the main methodological constraints of the previous research by exploring if wealth at the age of 37–38 predicts mortality up to age 62. The research design employed mitigates the problem of unobserved heterogeneity by using sibling and twin fixed-effects models.

**Findings:**

Both Kaplan–Meier survival analyses and the Cox proportional hazard regression results suggest that wealth is an important predictor of mortality even after individuals' observed and unobserved characteristics are accounted for with hazard ratios of 2.39 [95% confidence interval, CI 2.02, 2.83] among men and 1.74 [95% CI 1.39, 2.16] among women for the inverted cumulative density rank coefficients. The most disadvantaged groups are non-partnered men and women at the lower end of wealth distribution. Twin analyses align with the findings for the general population, indicating that wealth's effect on mortality is not confounded by genetic or shared family background factors.

**Interpretation:**

Our findings suggest that wealth is an important predictor of mortality, even in an advanced welfare state with comprehensive healthcare provisions, highlighting the need to address inequalities in wealth distribution to improve population health outcomes.

**Funding:**

The 10.13039/501100005416Research Council of Norway.

**Research in context**

**Evidence before this study**

We searched PubMed and grey literature through Google Scholar from January 1, 2004, to December 31, 2023. Our search terms included a combination of: “Wealth”, AND “mortality”, OR “all-cause mortality”, OR “cause-specific mortality”, OR “survival”, OR “life expectancy”. We also reviewed the reference lists of the selected articles to identify any further relevant studies. Evidence suggests that mortality disparity by wealth can be larger than inequalities by education, occupation, income, or childhood socioeconomic position. The main methodological limitation in this area of scholarship is its inability to account for individuals' unobserved heterogeneity, such as personality and genetic factors, which could potentially affect both their wealth level and survival probabilities. A few studies aim to address these concerns by examining the impact of sudden changes in wealth on mortality. This is done by analysing fluctuations in stock markets or lottery winnings as an external source of increased or decreased wealth. The latter designs have faced criticism for not accounting for self-selection into non-traditional wealth acquisition channels.

**Added value of this study**

It has been challenging to produce conclusive evidence on the links between wealth and mortality because being wealthy is associated with other individual traits that are difficult to measure, while self-reported information on wealth in survey data is often unreliable. Using register data from Norway, we could mitigate these concerns by analysing data for the entire population of Norway and studying the mortality outcomes of twins who differed from each other by their wealth levels. The significant role of wealth in explaining mortality outcomes within an advanced welfare state, where the impact of wealth on mortality is expected to be attenuated, indicates that wealth and its associated benefits are important for mortality even in societies characterized by the high quality of life, low income inequality, and extensive healthcare provisions.

**Implications of all the available evidence**

The results of this study indicate that increasing wealth inequality could worsen existing socioeconomic disparities in mortality in Western welfare democracies. Implementing policies aimed at levelling wealth distribution in Norway and beyond, among other areas in the housing market, is likely to positively affect the population's overall health.

## Introduction

There is ample evidence of the socioeconomic gradient in mortality. The studies conducted in different countries, settings, and periods demonstrate that individuals at the high end of the socioeconomic hierarchy have better health outcomes and live longer.^1^ One of the key mechanisms linking socioeconomic position with mortality might be the resources that individuals can deploy in various forms to maintain and improve their health.^2^ These resources take material (e.g., savings, property) and immaterial (e.g., knowledge, beneficial social connections) forms. Material resources in empirical studies on the socioeconomic determinants of mortality are most often operationalized through earnings (money earned from employment) or income (total money received).^3^ Yet, for a significant share of individuals, income represents only a fraction of the overall material resources they control.^4^ For most individuals, wealth is the central component of material resources, and there is a growing literature that focuses on wealth-based differentials in health, among other contexts, in low- and middle-income countries using innovative data collection techniques.^5^

Wealth can be described as abundant, valuable material possessions inherited, accumulated, saved, or acquired via other means.^6^ The major component of wealth for most individuals in Western welfare democracies is the housing they own.^7^ The better-quality housing that wealthier individuals can afford directly contributes to longer lifespans by providing better living standards, such as properly functioning heating and air conditioning systems, avoidance of overcrowded housing, and maintenance of a damp- and mold-free environment.^8^ More valued housing is usually located in districts and neighborhoods that are greener, are exposed to less noise, fumes, dust, and pollution from traffic and industry, have better infrastructure and lower levels of crime, and have access to recreational and sports facilities.^9^ In addition, lifestyle differences that are often associated with wealth, such as smoking, alcohol consumption, diet, and physical activity, are well-established mechanisms through which socioeconomic status influences health outcomes and mortality. Individuals with greater wealth are more likely to engage in healthier behaviors and have better access to resources that promote well-being.^10^

Further, theories of health inequality suggest that not only does the absolute amount of valuable possessions affect health but also that the relative position of individuals in the socioeconomic hierarchy matters for their likelihood of dying.^11^ The feelings of inequality and subordination among those at the lower end of the wealth hierarchy can result in the deterioration of physical health through biological mechanisms.^12^ Inequality-induced stressful situations may, for instance, trigger stimulation of the sympathetic nervous system with the resultant release of noradrenaline, rising heart rate and blood pressure, and narrowing of blood capillaries, potentially leading to deteriorating physical health.^13^ Social psychology scholarship also suggests that individuals are likely to compare their position with those of others, which can affect their health and well-being.^14^ It can be argued that wealth is a more explicit and demonstrative measure of socioeconomic position than income and plays a salient role in socioeconomic comparisons.

Scholars have already investigated how wealth is associated with mortality and longevity.^15^, ^16^, ^17^, ^18^ Some of these studies reveal that mortality disparity by wealth is larger than inequalities by education, occupation, income, or childhood socioeconomic position.^19^^,^^20^ The main methodological limitation in this area of scholarship is its inability to account for individuals' unobserved heterogeneity, such as personality and genetic factors, which could potentially affect both their wealth level and survival probabilities. A few studies try to mitigate these concerns by investigating the effect of wealth shocks on mortality by looking at stock market changes or lottery prizes as an exogenous source of increases or decreases in wealth.^21^^,^^22^ The designs of these studies, however, have been criticized due to self-selection into the less conventional channels of wealth acquisition.^23^

Further, when investigating the described association, an overlooked dimension is individuals' partnership status, which has close links with wealth and mortality. One of the central claims of classic sociological theory is that marriage provides individuals with an adult social role, a sense of meaning and purpose, and socio-emotional support, which directly affects their health and well-being outcomes.^24^ In contemporary settings, two equivalents of marriage, at least in Northern European societies, are partnership and cohabitation, which are also closely associated with the level of wealth and its accumulation.^25^ Evidence suggests that being non-partnered or experiencing union dissolution is linked to a lower level of wealth for both men and women. Still, women are particularly disadvantaged because men maintain their initial greater ownership and control over wealth.^26^ Being without a partner is independently linked to higher mortality, which can intensify the adverse effects of having low levels of wealth.

Additionally, the measures of wealth used in most existing research rely on individuals' self-reporting, which is known to be biased and does not capture the full extent of resources. This study addresses key methodological limitations in previous research and aims to examine whether a more precise measure of wealth, derived from the national register in Norway, serves as a significant predictor of mortality up to age 62 among partnered and non-partnered men and women. In our research design, we are able to account for the effects of family of upbringing and genetic characteristics by analyzing the effect of wealth on mortality in the population of twins. In addition to being a country with high-quality administrative data, Norway is a unique case for studying the link between wealth and mortality because it is one of the most advanced welfare states in the world, with universal healthcare provision and high living standards in which the influence of wealth on mortality can be expected to be attenuated. Yet, in recent decades, wealth inequality has been rising,^27^ and Norway today qualifies as a highly unequal country by wealth.^28^

## Methods

### Data

We rely on Norwegian administrative data for the country's entire population as the source of analyses. The administrative data includes individual- and family-level information from censuses, educational, tax, and income registers, and consists of birth cohorts from 1955 to 2000. However, since wealth data are available only for 1993–2017, our analytical selection is more restricted and contains birth cohorts born in 21 years between 1955 and 1975, with 620,282 men and 583,245 women (Table 1). Due to the limited availability of wealth data, at a maximum, the analyses track individuals from age 38 until 62, the late-middle age, or until their age of death, except for subset analyses when we control for occupations at age 48 as the baseline. Individuals' gender is based on the binary legal gender classification used by the Norwegian authorities in the administrative records. The outcome event is recorded as whether death has occurred and as the time relative to the baseline.

**Table 1:** Descriptive statistics: the general population and the population of twins.

	General Population				Twin Population			
	Men		Women		Men		Women	
	Mean	SD	Mean	SD	Mean	SD	Mean	SD
**Wealth and earnings**								
Gross wealth (mill. NOK)	2.4	8.3	1.3	3.4	2.2	4.1	1.2	2
Net wealth (mill. NOK)	1.3	7.9	0.85	3.2	1.2	3.7	0.76	1.8
Debt (mill. NOK)	1.2	1.5	0.48	0.77	1.1	1.3	0.45	0.72
Finance capital (mill. NOK)	0.36	7	0.18	2.5	0.29	2	0.15	1.2
Real capital (mill. NOK)	2.1	3	1.2	1.9	2	2.8	1.1	1.5
Gross wealth (cdr)	0.61	0.27	0.45	0.26	0.6	0.27	0.44	0.25
Net wealth (rank)	0.54	0.32	0.5	0.26	0.53	0.31	0.49	0.26
Earnings (100 K NOK)	5.1	3.7	3.3	1.9	5	2.8	3.2	1.8
Earnings (cdr)	0.64	0.26	0.39	0.25	0.64	0.26	0.39	0.25
**Immgrant background**								
No imm. background	0.94	0.24	0.94	0.24	0.94	0.25	0.94	0.25
Immigrant	0.009	0.095	0.008	0.09	0.008	0.089	0.006	0.078
Descendant	0.002	0.044	0.002	0.044	0.003	0.054	0.001	0.038
Other immigrant background	0.05	0.22	0.052	0.22	0.054	0.23	0.057	0.23
**Family composition**								
Birth order	2.1	1.2	2.1	1.2	3.3	1.3	3.3	1.3
Number of siblings	2.2	1.4	2.2	1.4	2.9	1.5	2.9	1.5
**Partnership status**								
Nonpartnered	0.52	0.5	0.45	0.5	0.53	0.5	0.46	0.5
Partnered	0.48	0.5	0.55	0.5	0.47	0.5	0.54	0.5
**Highest Education**								
Education (yrs)	12	2.9	13	2.9	12	2.9	12	2.9
Lower Secondary	0.36	0.48	0.39	0.49	0.37	0.48	0.42	0.49
Upper Secondary	0.37	0.48	0.27	0.44	0.36	0.48	0.26	0.44
Lower Tertiary (BA)	0.2	0.4	0.29	0.45	0.18	0.39	0.27	0.44
Higher tertiary (MA/PhD)	0.079	0.27	0.053	0.22	0.083	0.28	0.05	0.22
Observations	620,282		583,245		11,505		11,128	

Note: The immigrant population is low in the analytical selection: descendants are too young, the immigrants lack parental ID.

### Operationalizing of wealth and other sociodemographic and socioeconomic variables

The primary explanatory variable is wealth. Our preferred measure is gross wealth, estimated at the baseline age of 37–38. We use two years to derive information on wealth to mitigate the potential concerns of random variation in this variable measure from using only a single year. Gross wealth comprises all financial assets, real capital, and bank deposits. Wealth is adjusted to the consumer price index (CPI) and relativized to the birth year, both as deciles (divided into ten equal parts) and as an inverted cumulative density rank. The relativization of the wealth distributions makes it easier to compare effect sizes across time. We use the standard approach of estimating inverted cumulative density function rank that ranges from 0 (highest wealth) to 1 (lowest wealth).^29^ This operationalization accounts for the relative position of individuals in the wealth distribution and reduces the influence of extreme values possessed by the rich.^30^ We inverted the cumulative density rank measure of wealth to ensure that individuals with lower wealth correspond to positive hazard ratios for mortality, allowing for a more intuitive interpretation of the relationship between wealth and mortality risk.

All control variables, except the individuals' debt and labor market earnings, are measured at the baseline age of 37. These variables include the highest attained educational level, immigrant background, partnership status, birth order, and number of siblings. Individuals' total debt is the sum of the debt and loans that they owe to any financial institution domestically and abroad, including secured and unsecured mortgage debt (which constitutes the largest portion of debt in the country), consumer loans, student loans, credit card debt, car loans, and other personal debt, averaged for age 37–38. Both wealth and debt data come from tax authorities, which collect this information automatically from financial institutions for taxation purposes. The amount of wealth determines how much wealth tax should be paid to the municipality and the central government. The levels of wealth tax are quite low and have not changed much over time.^31^

The earnings variable is operationalized in the same way as the wealth variable using inverted cumulative density rank and includes the sum of personal income from employment and personal income from a business averaged over ages 37 and 38. The immigrant background variable separates whether individuals have no immigrant background or are immigrants or descendants of two immigrants and a residual category consisting of foreign-born individuals with one Norwegian parent, Norwegian-born individuals with one foreign-born parent, and Norwegian-born individuals born abroad to Norwegian parents. The relatively small number of immigrants in our analyses is determined by the fact that only those immigrants are included who arrived in Norway before the age of 38, who did not move abroad after turning 38, and, more importantly, who have an ID number linking them to their mothers which is the main method how we account for sibling fixed-effects in our models. Partnership status is divided into having a partner (married/cohabitants with common children) and non-partners (singles, divorcees, widows, and cohabitants without children). The fact that cohabitants without children are not identified in the administrative registers represents a limitation, and our results could potentially underestimate the differences between partners and non-partners. Birth order and number of siblings are measured as quantitative variables, although they are discrete states and not continuous. Descriptive statistics for the general and twin populations are presented in Table 1, while the same statistics without listwise deletion procedure are shown in the Supplementary Information, Table S1.

### Statistical analyses

The study relies on an event history analytical approach suitable for exploring how significant individuals' wealth is measured at certain life course stages to predict mortality outcomes at the end of the observation period. The analyses include descriptive Kaplan–Meier survivor graphs with wealth deciles and Cox proportional hazards regression models with the inverted cumulative density rank. In line with previous research, we conduct analyses separately for men and women. Within the Cox regression framework, we adopt three modeling strategies: (1) accounting for potential observable confounders, (2) conducting sibling fixed-effects analyses for the general population, and (3) performing twin fixed-effects analyses for the population of twin siblings.

The first strategy examines whether observable characteristics, such as debt, earnings, education, number of siblings, and birth order, can account for the mortality disparities between individuals of varying affluence levels. The second strategy additionally controls for all characteristics siblings share in the same family of upbringing, including unobservable characteristics. Such shared characteristics range from easily measurable factors such as social class backgrounds to more hard-to-measure characteristics such as the effects of living in the same neighborhoods, going to the same schools, and being exposed to particular parenting behaviors, styles, and norms. This methodological approach reduces the analyzed population as only individuals who have siblings are included in the analyses. In addition to the previous two strategies, the third strategy takes into account the potential differences that may exist among siblings who grew up in different environments within the same family. This approach is even more rigorous in controlling for the various behavioral and health tendencies that twins share due to their social and genetic background, which can be difficult to account for using traditional methods and may obscure the association between wealth and mortality.

The central proportional hazard assumption made by the Cox regression model is sometimes violated. But other models are faced with greater difficulties because the effect of time on the hazard is likely to be contaminated by unobserved sources of heterogeneity, which is crucial for our design.^32^ We assessed the proportional hazard assumption in the Cox regression model in several ways. First, we fitted alternative models, such as Weibull and exponential models, which account for changes in the hazard over time. These alternative model specifications yielded results consistent with those from the Cox regression model. We also did visual assessments of the proportional hazard assumption. Figure S1 in the Supplementary Information provides a visual representation of the Schönfeld residuals, based on the full Cox regression models, for wealth among both men and women, with none of the plots displaying systematic trends over time. Figure S2 presents a log–log plot for men and women across three selected groups of wealth deciles. While the lines for the groups are not perfectly straight, they do not cross or diverge significantly, suggesting that there is no severe violation of the proportional hazards assumption for these groups. Additionally, the good fit between the predicted and observed survival rates shown in Figure S3 further supports that the proportional hazards assumption holds for the groups in our descriptive Kaplan–Meier analyses. All data analyses are carried out using Stata 18 statistical software.

### Role of the funding source

The study sponsor, the Research Council of Norway (project number 275249), did not play any role in the study design, in the access, analyses, and interpretation of data, in the writing of the report, and in the decision to submit the study for publication.

## Results

### Kaplan–Meier survival analyses

**Fig. 1:**
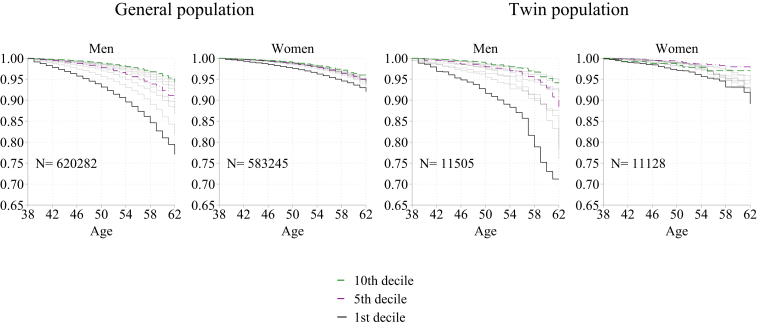
Kaplan–Meier survival analysis. Mortality according to gross wealth in deciles. 1st, 5th and 10th deciles highlighted. Men and women in the general and twin populations.

In Fig. 1, we first present results from Kaplan–Meier survival analyses for the deciles of wealth estimated separately for Norway's general population and twin population. We see that the overall pattern of association between wealth and mortality is similar in these two populations. The survival rates of men at the end of the analytical period are lower than those of women, as shown in the Supplementary Information, Figure S4, despite men being, on average, wealthier than women, as shown in Table 1 and the Supplementary Information, Figure S5. Yet, among the wealthiest individuals in the country, men and women in the ninth and tenth deciles of wealth distribution are very similar, with around 95% surviving up to age 62. The inequality in mortality is primarily observed between the middle and the bottom deciles of wealth, in general, and in the twin populations of men. After 24 years since the estimation of individuals' wealth, around 90% of men are alive in the fifth wealth decile, while in the first wealth decile, the share of surviving men is less than 80%. No corresponding wealth inequality in mortality is observed among women.

**Fig. 2:**
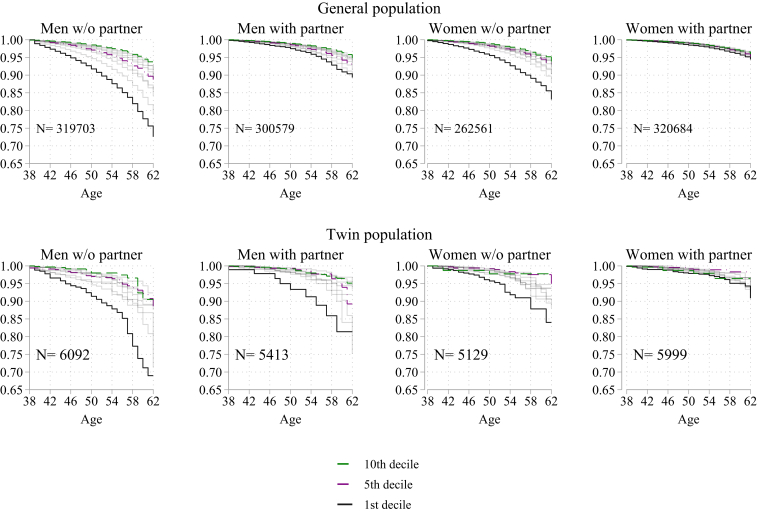
Kaplan–Meier: Survival rates according to wealth, marital status and gender.

In the Supplementary Information, Figure S6, we see a large mortality disparity between partnered and non-partnered (nonmarried/divorced/widowed) individuals. In Fig. 2, we present the results from the Kaplan–Meier survival analyses for individuals in the general and twin populations separately for partnered and non-partnered individuals. The main finding from these analyses is that wealth differences in survival rates are more substantial for non-partnered than partnered individuals. Further, the results suggest that the gender gap in survival rates is related to partnership status. Among women without a partner, the wealth differences in mortality are more in line with the results for men, although wealth inequality is still smaller for women than for men. For non-partnered individuals, less than 75% of men are alive in the first wealth decile up to age 62, while the share of surviving women is less than 85%. The health inequalities among partnered men are small (around five percentage points between those in the top and the bottom wealth deciles) and nearly nonexistent between partnered women with the most and the least wealth. Among partnered individuals, about 90% of men and 95% of women are alive in the first wealth decile after 24 years. Similar patterns of associations between wealth, gender, and partnership status are also observed in the twin population.

The Kaplan–Meier survival analyses in the Supplementary Information, Figures S7–S12, indicate that, in addition to wealth, gender, and partnership status, the mortality risk in Norway is stratified by whether or not individuals have debt, and their labor market earnings, education, migration background, birth order, and number of siblings. By the end of the observation period, the survival rates are higher among men and women with high earnings, a university education, an immigration background, and siblings, and those who are firstborn in the family. These characteristics are also associated with wealth, as shown in the Supplementary Information, Table S2, and are accounted for in Cox proportional models. When exploring mortality inequality by individuals' indebtedness in the Supplementary Information, Figure S7, we have identified that those with the highest debt levels do not have the lowest survival rates by the end of the observation period. The latter suggests that using the measure of net wealth (defined as the total value of all assets owned by the individual minus any liabilities such as debts and loans) in mortality estimations could produce measurement bias because individuals with the lowest level of debt have higher mortality risk than some groups of individuals with more debt. This indicates that individuals' debt should be non-linearly accounted for when analyzing the links between wealth and mortality.

### Cox proportional hazard models

Tables 2 and 3 present results from Cox proportional hazard models. The models consecutively account for wealth only, adding stepwise individuals' debt and its squared term, earnings, and sociodemographic characteristics such as birth order, number of siblings, migration background, education, and partnership status. In contrast, the final model additionally accounts for sibling fixed-effects. For the twin population, we have the same model specifications. Those characteristics that are identical for twins, such as birth order, are excluded, while in the final model, we substitute sibling fixed-effects with twin fixed-effects.

**Table 2:** Cox regression models: Gross wealth. Inverted Cumulative Density Ranks (CDR). General population.

	Men					Women				
	(1)	(2)	(3)	(4)	(5)	(6)	(7)	(8)	(9)	(10)
	Base	Debt	Earnings	Controls	Fam-FE	Base	Debt	Earnings	Controls	Fam-FE
**Financial situation**										
Gross wealth (cdr)	5.76∗∗∗	5.30∗∗∗	3.26∗∗∗	2.66∗∗∗	2.39∗∗∗	1.56∗∗∗	1.88∗∗∗	1.59∗∗∗	1.64∗∗∗	1.74∗∗∗
	[5.47, 6.06]	[5.01, 5.60]	[3.07, 3.46]	[2.50, 2.82]	[2.02, 2.83]	[1.45, 1.69]	[1.72, 2.05]	[1.46, 1.74]	[1.50, 1.80]	[1.39, 2.16]
Debt (mill. NOK)		0.93∗∗∗	0.99	1.02	1.06		1.39∗∗∗	1.62∗∗∗	1.22∗∗∗	1.31∗∗
		[0.91, 0.95]	[0.97, 1.01]	[0.99, 1.04]	[0.98, 1.14]		[1.28, 1.50]	[1.49, 1.75]	[1.13, 1.31]	[1.10, 1.56]
Debt (mill. NOK) # Debt (mill. NOK)		1.00∗∗∗	1.00	1.00	1.00		0.91∗∗∗	0.89∗∗∗	0.97∗∗	0.94∗
		[1.00, 1.00]	[1.00, 1.00]	[1.00, 1.00]	[0.99, 1.01]		[0.88, 0.94]	[0.86, 0.92]	[0.94, 0.99]	[0.88, 1.00]
Earnings (cdr)			3.68∗∗∗	2.90∗∗∗	2.88∗∗∗			2.44∗∗∗	1.93∗∗∗	1.95∗∗∗
			[3.48, 3.89]	[2.73, 3.07]	[2.48, 3.34]			[2.24, 2.67]	[1.76, 2.12]	[1.58, 2.40]
**Highest Education**										
Lower Secondary				1.00	1.00				1.00	1.00
				[1.00, 1.00]	[1.00, 1.00]				[1.00, 1.00]	[1.00, 1.00]
Upper Secondary				0.77∗∗∗	0.79∗∗∗				0.76∗∗∗	0.76∗∗∗
				[0.74, 0.80]	[0.73, 0.87]				[0.72, 0.79]	[0.67, 0.86]
Lower Tertiary (BA)				0.66∗∗∗	0.71∗∗∗				0.68∗∗∗	0.72∗∗∗
				[0.63, 0.69]	[0.62, 0.81]				[0.64, 0.71]	[0.62, 0.84]
Higher tertiary (MA/PhD)				0.66∗∗∗	0.78∗				0.70∗∗∗	0.70∗
				[0.61, 0.72]	[0.63, 0.96]				[0.61, 0.79]	[0.51, 0.97]
**Partnership status**										
Nonpartnered				1.46∗∗∗	1.44∗∗∗				1.68∗∗∗	1.48∗∗∗
				[1.41, 1.51]	[1.33, 1.56]				[1.62, 1.75]	[1.34, 1.63]
Partnered				1.00	1.00				1.00	1.00
				[1.00, 1.00]	[1.00, 1.00]				[1.00, 1.00]	[1.00, 1.00]
**Family composition**										
Birth order				1.00	0.99				1.00	1.01
				[0.99, 1.02]	[0.96, 1.02]				[0.98, 1.02]	[0.98, 1.06]
Number of siblings				0.95∗∗∗					0.96∗∗∗	
				[0.94, 0.97]					[0.95, 0.98]	
**Immigrant background**										
No imm. background				1.00					1.00	
				[1.00, 1.00]					[1.00, 1.00]	
Immigrant				0.84					0.95	
				[0.69, 1.02]					[0.72, 1.25]	
Descendant				1.09					1.12	
				[0.77, 1.54]					[0.73, 1.72]	
Other				1.05					1.04	
				[0.98, 1.12]					[0.95, 1.13]	
Observations	620,282	620,282	620,282	620,282	620,282	583,245	583,245	583,245	583,245	583,245

Exponentiated coefficients; 95% confidence intervals in brackets.

∗*p* < .05.

∗∗*p* < .01.

∗∗∗*p* < .001.

**Table 3:** Cox regression models: Gross wealth. Inverted Cumulative Density Ranks (CDR). Twin population.

	Men					Women				
	(1)	(2)	(3)	(4)	(5)	(6)	(7)	(8)	(9)	(10)
	Base	Debt	Earnings	Controls	Twin-FE	Base	Debt	Earnings	Controls	Twin-FE
**Financial situation**										
Gross wealth (cdr)	4.81∗∗∗	4.46∗∗∗	2.54∗∗∗	2.09∗∗∗	2.78	1.29	1.81	1.55	1.62	2.57
	[3.32, 6.96]	[2.99, 6.66]	[1.67, 3.87]	[1.35, 3.23]	[0.94, 8.27]	[0.76, 2.18]	[0.98, 3.33]	[0.83, 2.89]	[0.85, 3.06]	[0.58, 11.47]
Debt (mill. NOK)		0.92	1.01	1.03	0.96		1.70	1.95∗	1.62	1.91
		[0.78, 1.08]	[0.86, 1.18]	[0.88, 1.20]	[0.45, 2.01]		[0.98, 2.94]	[1.10, 3.44]	[0.94, 2.79]	[0.49, 7.38]
Debt (mill. NOK) # Debt (mill. NOK)		1.00	1.00	1.00	1.01		0.88	0.86	0.92	0.97
		[1.00, 1.01]	[1.00, 1.01]	[0.99, 1.01]	[0.83, 1.23]		[0.70, 1.11]	[0.67, 1.09]	[0.75, 1.13]	[0.56, 1.69]
Earnings (cdr)			4.48∗∗∗	3.70∗∗∗	2.63			2.13∗	1.68	1.19
			[2.98, 6.75]	[2.42, 5.67]	[0.92, 7.48]			[1.17, 3.85]	[0.89, 3.17]	[0.28, 5.05]
**Highest Education**										
Lower Secondary				1.00	1.00				1.00	1.00
				[1.00, 1.00]	[1.00, 1.00]				[1.00, 1.00]	[1.00, 1.00]
Upper Secondary				0.82	0.88				0.66∗	0.41
				[0.64, 1.06]	[0.48, 1.60]				[0.46, 0.94]	[0.14, 1.18]
Lower Tertiary (BA)				0.83	0.73				0.73	0.37
				[0.59, 1.16]	[0.30, 1.74]				[0.51, 1.04]	[0.08, 1.71]
Higher tertiary (MA/PhD)				0.61	0.00				0.58	0.98
				[0.33, 1.13]	[0.00, 0.00]				[0.23, 1.46]	[0.06, 16.93]
**Partnership status**										
Nonpartnered				1.45∗∗	1.18				1.45∗∗	1.13
				[1.15, 1.83]	[0.68, 2.04]				[1.11, 1.91]	[0.62, 2.08]
Partnered				1.00	1.00				1.00	1.00
				[1.00, 1.00]	[1.00, 1.00]				[1.00, 1.00]	[1.00, 1.00]
**Family composition**										
Number of siblings				0.96					0.97	
				[0.90, 1.03]					[0.89, 1.06]	
**Immigrant background**										
No imm. background				1.00					1.00	
				[1.00, 1.00]					[1.00, 1.00]	
Immigrant				1.19					0.00	
				[0.30, 4.81]					[0.00, 0.00]	
Descendant				2.15					3.82	
				[0.53, 8.67]					[0.53, 27.38]	
Other				0.52∗					1.18	
				[0.28, 0.98]					[0.68, 2.02]	
Observations	11,505	11,505	11,505	11,505	11,505	11,128	11,128	11,128	11,128	11,128

Exponentiated coefficients; 95% confidence intervals in brackets.

∗ *p* < .05.

∗∗*p* < .01.

∗∗∗*p* < .001.

The inverted cumulative density rank coefficients indicate the difference in the outcome measure between individuals in the first and top percentiles of wealth distribution. We see that in the general population of men, those in the lowest part of the wealth distribution have 5.8 [95% confidence interval (CI) 5.5, 6.1] times the hazard of dying than the most affluent men. This effect size is reduced when individuals' debt is accounted for, but the hazard ratio of the wealth variable is almost halved to 3.3 [95% CI 3.1, 3.5] when the earnings variable is introduced in Cox regression. The effect size of the wealth variable for men is further reduced in the sibling fixed-effects model. For the general population of women, the effect size of the wealth variable is consistently smaller than for men. Still, it remains substantive after including sibling fixed-effects with a hazard ratio of 1.7 [95% CI 1.4, 2.2]. Interaction models of the pooled population in the Supplementary Information, Table S3, confirm that wealth is a more important predictor of mortality in men than women. To account for uneven mortality rates between men and women, in the Supplementary Information, Table S4, we present the estimates of men's mortality at age 57 and women's mortality at age 62. The results are qualitatively similar to the main estimates in Table 2.

The analyses of the population of twins show the same pattern of associations as in the results for the general population. After individuals' debt and earnings are included in Cox regressions, the hazard ratio for men is halved to 2.5 [95% CI 1.7, 3.9], while for twin women, the hazard ratio remains consistently lower with larger confidence intervals than for twin men. In the final model specification for the twin men population that accounts for twin fixed-effects, wealth is a significant predictor of mortality up to age 62 at the 10% significance level with a hazard ratio of 2.8 [95% CI 0.9, 8.3].

### Partnership status, wealth, and mortality

**Fig. 3:**
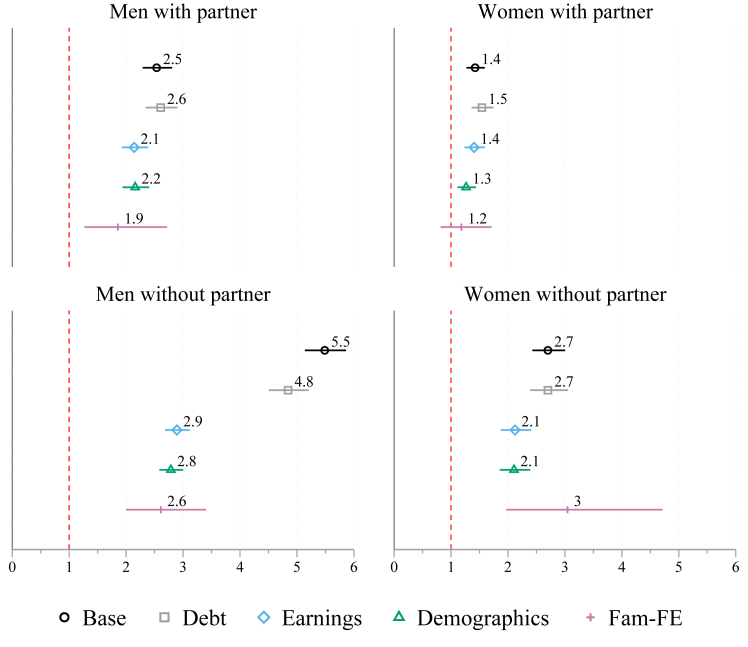
Hazard ratios of mortality according to gross wealth according to gender and marital status. Cox regression models on men and women in the general populations. 95% CI.

Fig. 3 shows results from Cox proportional hazard models according to partnership status in the general population. In line with the descriptive Kaplan–Meier analyses in Fig. 2, the results suggest that wealth is a stronger predictor of mortality among non-partnered than partnered individuals. This is formally confirmed with interaction models between wealth and partnership status in the Supplementary Information, Table S5. Regardless of gender, the effect sizes for partnered individuals are smaller. Among partnered men, the effect size of wealth is a hazard ratio of 2.5 [95% CI 2.3, 2.8], while it is 1.4 [95% CI 1.3, 1.6] among partnered women. Controlling for all considered variables and sibling fixed-effects, the effect size of the wealth variable for men is a hazard ratio of 1.9 [95% CI 1.3, 2.7] and 1.2 for women [95% CI 0.8, 1.7]. It is apparent that while wealth matters more for mortality among partnered men than partnered women, men and women without partners are very similar in terms of wealth disparities in mortality. Although the initial effect size of wealth is a hazard ratio of 5.5 [95% CI 5.1, 5.9] for men and 2.7 [95% CI 2.4, 3.0] for women, after controlling for debt, earnings, demographics, other controls, and sibling fixed-effects in Fig. 3 (for full details, see Table S6 in the Supplementary Information), the effect size of the wealth variable for men, with a hazard ratio of 2.6 [95% CI 2.0, 3.4], and women, with a hazard ratio of 3.0 [95% CI 2.0, 4.7], are close to each other. Table S7 in the Supplementary Information presents the results for the twin population, which are in line with the main findings for the country's general population, but the confidence intervals of these estimates are wider due to the low sample size of twins with varying partnership status.

### Further analyses of the links between wealth and mortality

We conducted additional analyses to explore potential mechanisms linking wealth and mortality and to check the robustness of the main findings. First, in the Supplementary Information, Figure S13 and Tables S8 and S9, we substitute the gross wealth variable used in the main analyses with the net wealth variable, but we do not include individuals' debt in this specification as the net wealth variable already accounts for debt. In the results for the twin men population, the net wealth variable loses its significance probably because this measure of wealth confounds debt and wealth. Debt has different meanings for different socioeconomic groups, as many individuals in Norway take up mortgages to enter the housing market in their late 20s and 30s. For high-earnings groups, high debts are likely to be converted to housing wealth, while for low-earnings groups, having debt is more likely to be related to consumption. The net wealth variable cannot account for such differences.^33^

**Fig. 4:**
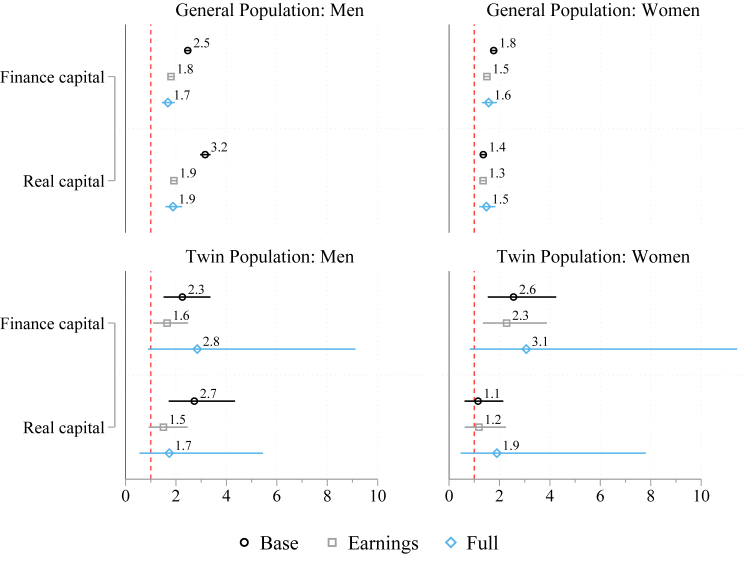
Hazard ratios of mortality according to financial and real capital. Cox regression models on men and women in the general and twin populations. 95% CI.

In the Supplementary Information, Figure S14 and Table S15, we present mortality hazard ratios by individuals' wealth quintiles. As seen in the Kaplan–Meier survival analyses, the significant effects of wealth on mortality in the general population are observed among those at the low end of wealth distribution. Hazard ratios of estimates with twin fixed-effects are comparable to those of the general population estimates with sibling fixed-effects, but confidence intervals are larger. To understand whether the different types of wealth have varying links with mortality, in Fig. 4, we differentiate between finance capital and real capital components of wealth operationalized in the same way as the main wealth variable with inverted cumulative density ranks. Both real and financial capital are important predictors of mortality. In the estimates for twin women, finance capital has consistently higher hazard ratios than real capital. The full results are shown in the Supplementary Information, Tables S11 and S12.

In the Supplementary Information, Tables S13, we explore the association between wealth and mortality in shorter follow-up periods of 5, 10, and 15 years. The results indicate that the effect of wealth on mortality becomes apparent after a 5-year follow-up period. In the Supplementary Information, Tables S14 and S15, we also consider the potential effects of living in Norway's five biggest cities, which have vibrant housing markets affecting household wealth. Still, this specification does little to change our estimates for the wealth variable. Finally, for a subset of our general population of Norway, for which data on occupational categories are available, in the Supplementary Information, Table S16, we include in our Cox proportional hazards regression models the detailed codes for the International Standard Classification of Occupations and observe that the association between wealth and mortality is not affected by the type of occupations that men and women have.

## Discussion

Insights from scholarship on socioeconomic determinants of mortality suggest that the wealth-enabled possession, acquisition, and consumption of certain material and immaterial goods and services benefit health and can prolong individuals' lives. From a socio-psychological perspective, perceived social position is a significant predictor of mortality, and wealth might be an important dimension through which individuals compare their standing in the socioeconomic hierarchy with others. However, due to constraints related to the quality of data on individuals' wealth, there is only limited evidence on this important area of research. One of the main methodological challenges is individuals' unobserved heterogeneity, which can be linked to wealth and mortality.^34^ A no less important concern for researching this topic is access to unbiased information on wealth, as individuals in survey data often provide distorted estimates of their wealth.^35^ We used data on Norway's general and twin populations with high-quality wealth information from the country's administrative registers to mitigate the described methodological constraints.

Our findings from Kaplan–Meier survival analyses and the Cox proportional hazards regression models reveal that wealth has an important role in predicting individuals' mortality by up to age 62. Wealth remains a substantially important variable after considering the effects of individual characteristics associated with mortality and wealth. These results are not significantly affected by accounting for the shared characteristics among siblings and twins, such as their genetic composition. In other words, the evidence from our study suggests that wealth's effect on mortality is not confounded by different aspects of socioeconomic position and unobserved characteristics fixed within families and among twins. One of the central findings of our study is that the association between wealth and mortality depends on individuals' gender. Wealth measured at age 37–38 matters more for men's survival chances up to age 62 than for women. Wealth is not a significant predictor of mortality among partnered women in sibling fixed-effects models; however, among non-partnered individuals, the effect of wealth is largely comparable between genders. There might be several complementary explanations for this finding.

First, the women's mortality rate in Norway, including among those who are non-partnered, is lower than the men's mortality rate, which leaves fewer possibilities to explain variation in mortality by wealth and other known determinants of mortality, yet wealth still might matter for women's morbidity outcomes. Second, on average, women have lower levels of wealth than men, which potentially reduces the overall importance of wealth for partnered women in various aspects of life, including in social comparison of status with its detrimental mortality consequences.^36^ Third, there might be important gender differences regarding personal and household wealth's role in mortality outcomes. Norway is one of the most gender-egalitarian countries in the world, yet, in partnerships, men are more likely than women to be owners of real and financial capital. This could imply that overall household wealth is more important for women than individual-level wealth used in our research.

Another central finding of this study is that the identified inequality is most salient among men situated in the lower strata of wealth distribution. Mortality differences are much higher between the middle and the low-wealth groups than between the top and middle-wealth groups. The profile of men with the least wealth by age 37–38 consists of those who do not own property and have few other assets. Our results suggest that both real and financial capital are important predictors of mortality by age 62. The end of the 30s is the stage of individuals' life course when they achieve maturity in various aspects of socioeconomic position. Individuals who, by the end of the fourth decade of their lives, are not able to accumulate any wealth are likely to have concurring negative life experiences such as being homeless, having fixed-term employment, or experiencing spells of long-term unemployment. One of our central findings that wealth is a more important predictor of mortality at the lower end of the distribution mirrors an association between earnings and mortality observed in Norway, suggesting that major inequality occurs between those in the bottom earnings group and the rest of the population.^37^

Methodologically, we argue that using gross wealth, as opposed to net wealth, provides a more accurate understanding of the relationship between wealth and mortality. Gross wealth includes the total value of an individual's assets without reducing it by subtracting debts. This offers a more complete picture of an individual's financial resources. In contrast, net wealth, which combines assets and debts into one figure, can obscure important differences between individuals who may have the same net wealth but vastly different financial situations. For example, debt, particularly secured debt like mortgages, often represents opportunities for wealth-building rather than financial hardship, and this distinction is lost when using net wealth alone. Moreover, net wealth can lead to misclassifications by grouping households with high assets and debt together with those with neither assets nor debt despite their vastly different economic realities. By considering gross wealth and debt separately, we avoid these oversimplifications and offer a clearer insight into the impact of wealth on mortality outcomes.^33^

Our study has several limitations. First, due to the unavailability of wealth data for cohorts born in the early 20th century, we cannot conduct analyses of individuals' survival by their wealth beyond the late-middle age of 62. This can be more problematic in relation to the mortality of women, who have longer life expectancy than the country's population of men. Besides, the most common causes of death in late-middle age are different from the most common causes of death in older ages, suggesting that different mechanisms may explain wealth inequalities in late-middle age mortality. While our study focused on mortality in late-middle age, we did not analyze mortality across different age groups or time periods. This limits our ability to generalize the findings to older or younger populations and to understand how the wealth–mortality relationship may change over time.

Second, it has been challenging to include partners' wealth in our analyses due to the period when wealth is measured and the age difference between men and women. For example, more women than men would be left-censored from the sample because they tend to have older partners. Conceptually, it is also unclear whether the level of a person's partner's wealth should be measured in terms of their own or the partner's age.

Third, despite employing high-quality register data and the population of twins in Norway, our research design does not allow us to eliminate all unobserved heterogeneity among individuals. There might be considerable differences between siblings in personality and lifestyle, which are likely to affect both wealth at the baseline age of 37–38 and the risk of mortality in late-middle age. Fourth, we did not examine cause-specific mortality in this study, which limits the scope of our conclusions regarding the pathways through which wealth influences mortality. Investigating specific causes of death, such as cardiovascular diseases or cancers, could provide further insights into the mechanisms linking wealth and health. Fifth, although we used comprehensive register data for the entire population of Norway, there was limited information on the immigrant population. Given that immigrants may experience different wealth trajectories and mortality risks, future research should explore how these dynamics differ across immigrant subgroups.

Additionally, as only monozygotic (MZ) twins are genetically identical, but the prevalence of dizygotic (DZ) twins is higher in a typical population, the employed twin fixed-effects strategy might also not eliminate unobserved heterogeneity related to individuals’ genetics. Therefore, we do not claim that we identify the causal effects of wealth on mortality. Nonetheless, including MZ and DZ twins in our study offers significant advantages over analyses relying on the general population. By using twin data, we are able to control for shared environmental and genetic factors more effectively, especially when employing fixed-effects models. Notably, in our gender-specific analysis, the proportion of MZ twins is higher compared to the pooled analysis of both genders. This increased representation of MZ twins provides an advantage, as MZ twins offer more precise control for genetic influences, allowing us to better isolate the impact of wealth on mortality. Another limitation is that we only measure wealth and other variables at the baseline age of 37–38. Wealth and other considered characteristics such as earnings, number of children, or occupational category are likely to change over life. Finally, although Norway offers a unique welfare context, generalizability to other European countries may be limited, particularly to nations with higher levels of wealth inequality or different healthcare systems. Further comparative research across European countries would help to clarify the broader applicability of these findings.

After discussing the results of our study and its methodological limitations, we can briefly elaborate on the potential implications of the findings for wealth and mortality inequality research. In light of the presented results, it is evident that the rise in wealth inequality could potentially amplify the existing socioeconomic gaps in mortality observed in many Western welfare democracies. The capacity of wealth to predict mortality outcomes in Norway highlights the significance of wealth and its associated benefits for health, even within a society characterized by a generally high quality of life, low income inequality, and comprehensive healthcare provisions. A particularly important component of overall wealth is housing, which has become increasingly unaffordable for young generations without their families' financial support and stable earnings.^38^ Implementing policies aimed at leveling wealth distribution in Norway and beyond, among other areas in the housing market, is likely to affect the population's overall health positively.

## Contributors

AG and ØNW conceptualized and designed the study. ØNW performed the data management and analyses. AG and ØNW drafted the manuscript. Both authors were involved in the preparation and review of the final manuscript.

## Data sharing statement

The data used in this study are from the national censuses and educational, tax, and income registers. Research with these data was approved by the Norwegian Agency for Shared Services in Education and Research. Access to the data is strictly regulated and was given to the present study's authors by Statistics Norway. These data were used under license for the authors' research project and are not publicly available. The data can become accessible to authorized researchers after the approval of a formal application. The response time for data applications varies by the demand and capacity of Statistics Norway. Depending on the circumstances, waiting times can range from a few months to up to two years. The replication code for this study is available in the OSF repository.^39^

## Declaration of interests

The authors have no conflicts of interest relevant to this study to disclose.
