# Supplementary material for: Wealth and mortality among late-middle-aged individuals in Norway: a nationwide register-based retrospective study
Source: Lancet Reg Health Eur. 2024 Nov 8;48:101113. doi: 10.1016/j.lanepe.2024.101113 (PMC11584759; doi:10.1016/j.lanepe.2024.101113)
Supplement: Supplementary Figures and Tables [file mmc1.pdf]

Wealth and mortality among late-middle-aged individuals in Norway: a nationwide register-based retrospective study

October 28, 2024

Contents

1 Supplementary Information: Figures and Tables **2**

List of Figures

S1 Wealth and mortality: KM-plot vs predicted survival rates from Cox regression 2
S2 Wealth and mortality: log-log plot 3
S3 Wealth and mortality: Schönfeld residuals from full Cox regression models with family fixed effects 3
S4 Kaplan-Meier: Survival rates according to gender 4
S5 Kernel density plots of ln(Gross Wealth) over gender 5
S6 Kaplan-Meier: Survival rates according to marital status and gender 5
S7 Kaplan-Meier: Survival rates according to debt and gender 6
S8 Kaplan-Meier: Survival rates according to earnings and gender 6
S9 Kaplan-Meier: Survival rates according to education level and gender 7
S10 Kaplan-Meier: Survival rates according to immigration background 7
S11 Kaplan-Meier: Survival rates according to birthorder and gender 8
S12 Kaplan-Meier: Survival rates according to number of siblings and gender 8
S13 Kaplan-Meier: Survival rates according to net wealth and gender 9
S14 Hazard ratios of mortality according to gross wealth as quintiles. Cox regression models on males and females in the general and twin populations. 95%-Confidence Intervals. . . . 9

List of Tables

S1 Descriptive statistics without listwise deletion: the general population and the population of twins. Immigrants arriving after the age of 38 or nonresidents at the same age are removed. Most missingness are due to lack of family/parental and educational information of immigrants. 10
S2 Correlation matrix: the general population 11
S3 Cox regression models: Gross wealth X Gender interactions tested 12
S4 Cox regression models: Gross wealth. Inverted Cumulative Density Ranks (CDR). General population. Robustness checks of different time restrictions according to gender: males observed for 20 years, females for 25 years. Compare with Table 2 13
S5 Cox regression models: Gross wealth X Partnership interactions tested 14
S6 Cox regression models: Partnership status - Gross wealth. Inverted Cumulative Density Ranks (CDR). General population. Underlying Figure 3 15
S7 Cox regression models: Partnership status - Gross wealth. Inverted Cumulative Density Ranks (CDR). Twin population. 16
S8 Cox regression models: Net wealth. Inverted Cumulative Density Ranks (CDR). General population. 17
S9 Cox regression models: Net wealth. Inverted Cumulative Density Ranks (CDR). Twin population. 18

S10 Cox regression models: Gross wealth. Quintiles. 19
S11 Cox regression models: Finance and real capital. Inverted Cumulative Density Ranks (CDR). General population. 20
S12 Cox regression models: Finance and real capital. Inverted Cumulative Density Ranks (CDR). Twin population. 21
S13 Cox regression models: Gross wealth. Inverted Cumulative Density Ranks (CDR). General population. Robustness checks of different time restrictions:Time in 5,10,15. Compare with Table 2 22
S14 Cox regression models: Gross wealth. Inverted Cumulative Density Ranks (CDR). General population. Models controlled for high density cities 23
S15 Cox regression models: Gross wealth. Inverted Cumulative Density Ranks (CDR). Twin population. Models controlled for high density cities 24
S16 Cox regression models: Gross wealth. Inverted Cumulative Density Ranks (CDR). General population. Controlled for 1 and 4 digits occupational ISCO-88 codes 25

1 Supplementary Information: Figures and Tables

Figure S1: Wealth and mortality: KM-plot vs predicted survival rates from Cox regression

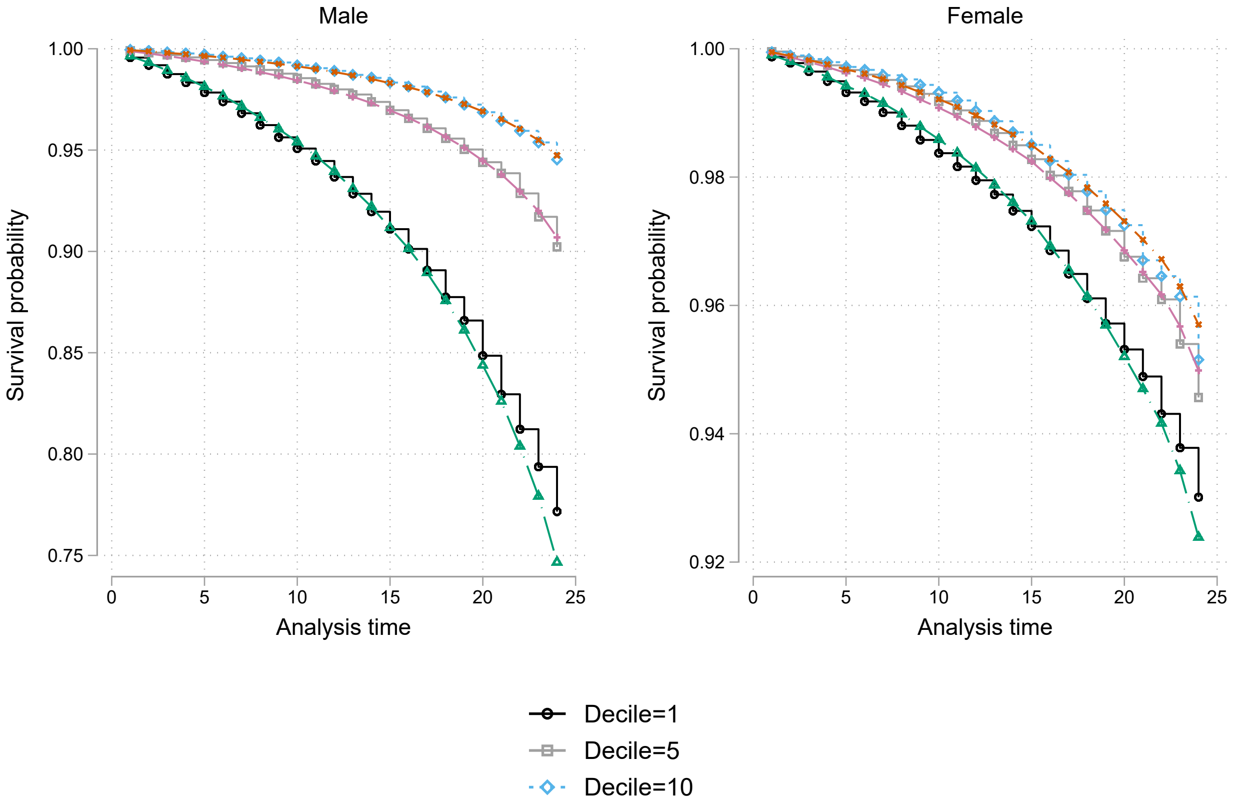

Figure S2: Wealth and mortality: log-log plot

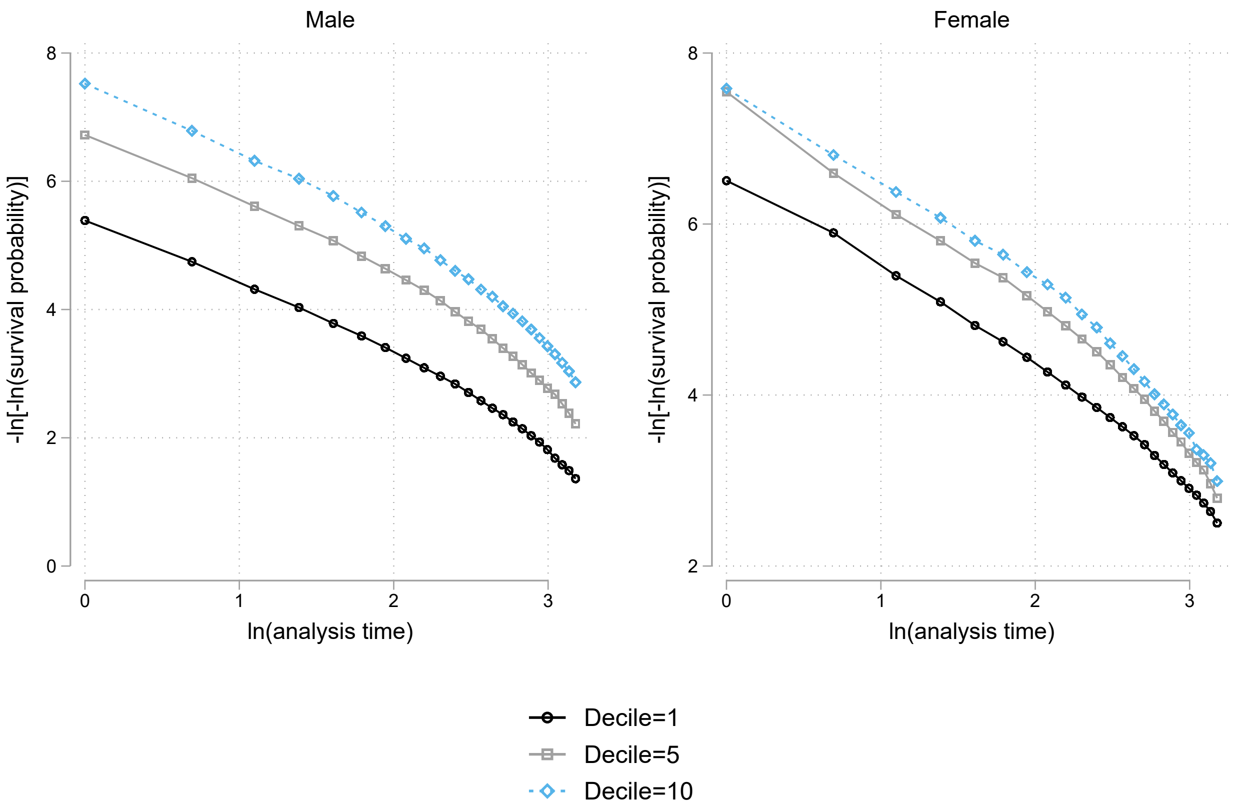

Figure S3: Wealth and mortality: Schönfeld residuals from full Cox regression models with family fixed effects

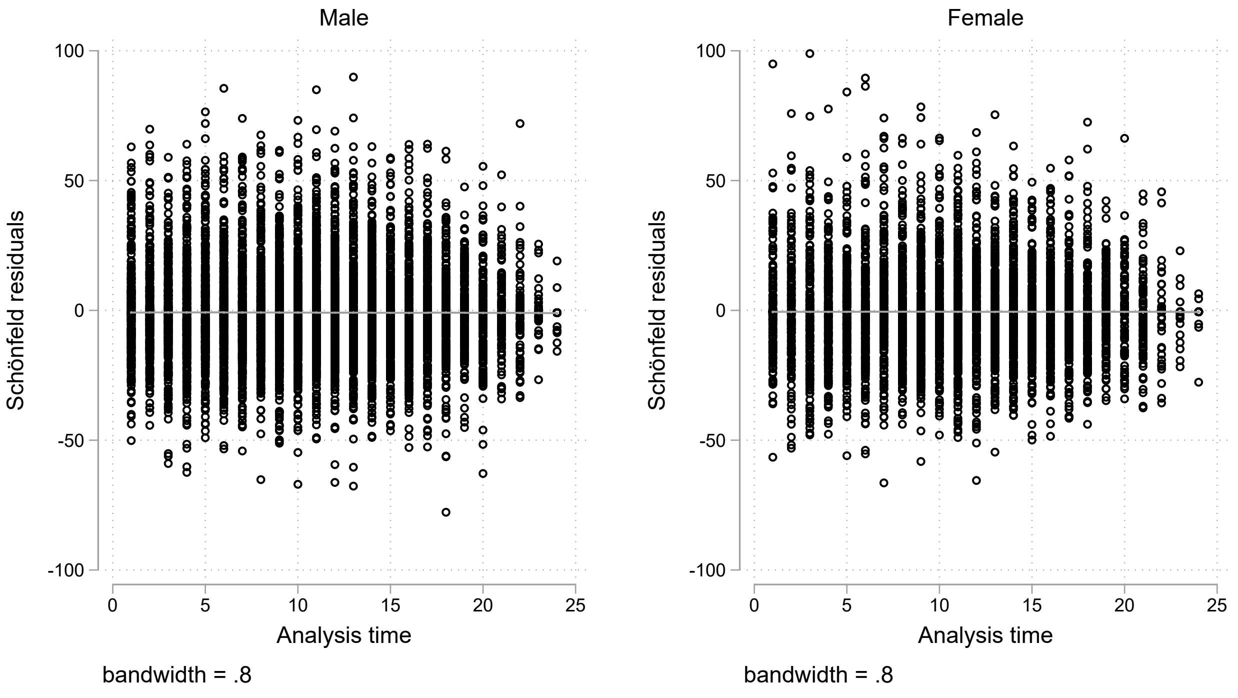

Figure S4: Kaplan-Meier: Survival rates according to gender

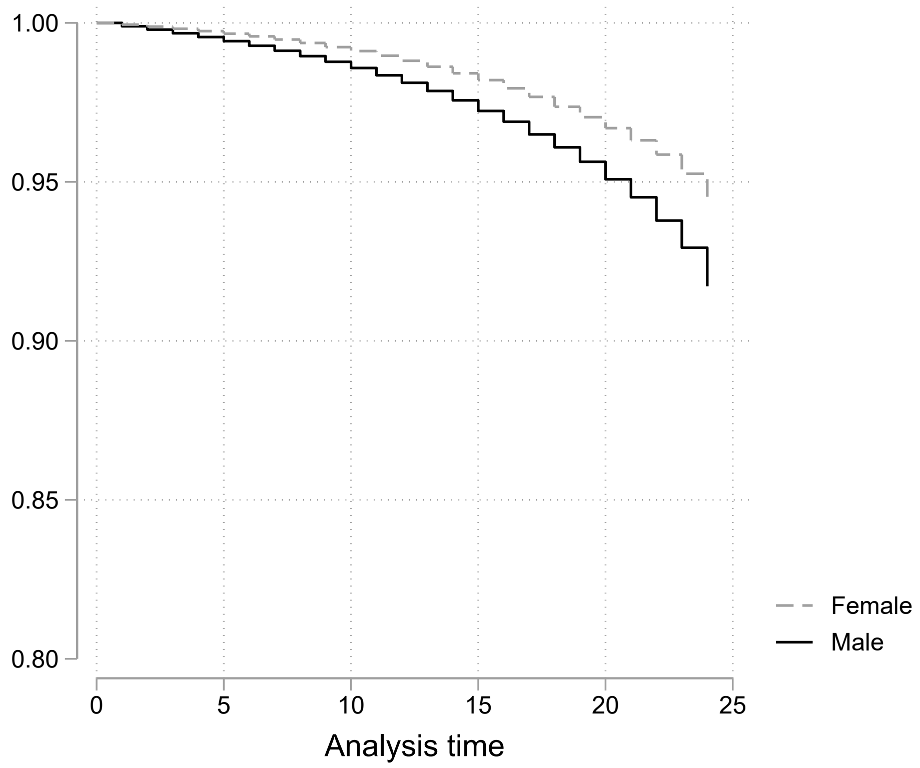

Figure S5: Kernel density plots of ln(Gross Wealth) over gender

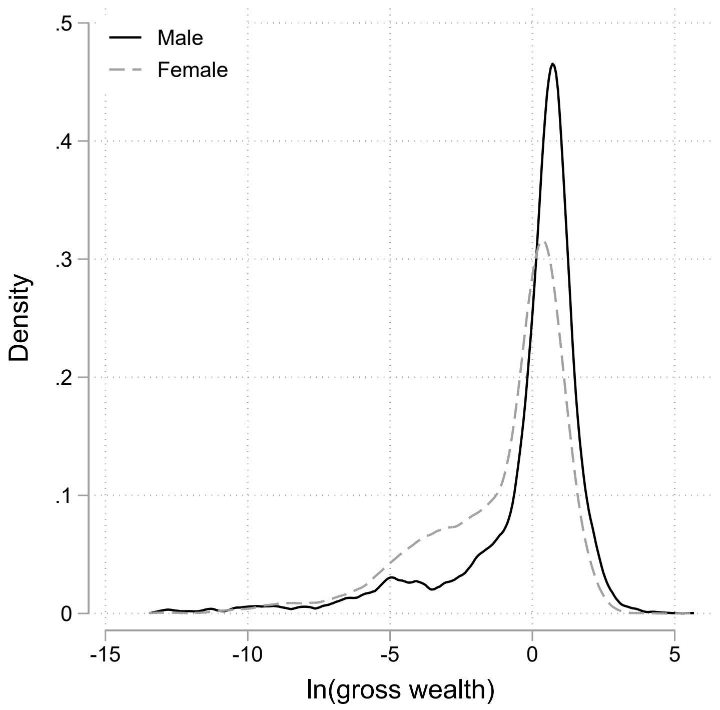

Figure S6: Kaplan-Meier: Survival rates according to marital status and gender

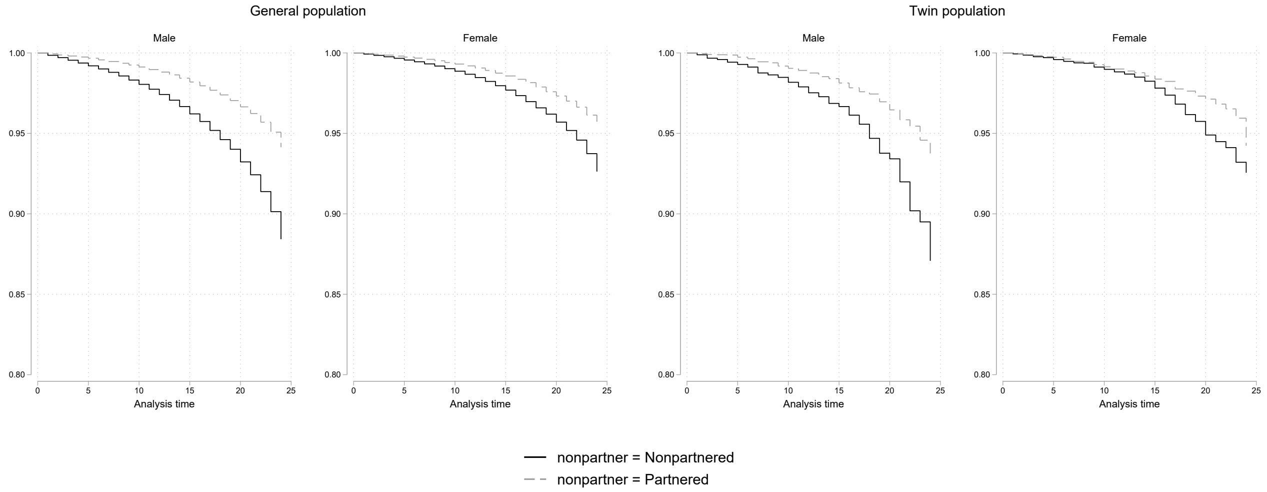

Figure S7: Kaplan-Meier: Survival rates according to debt and gender

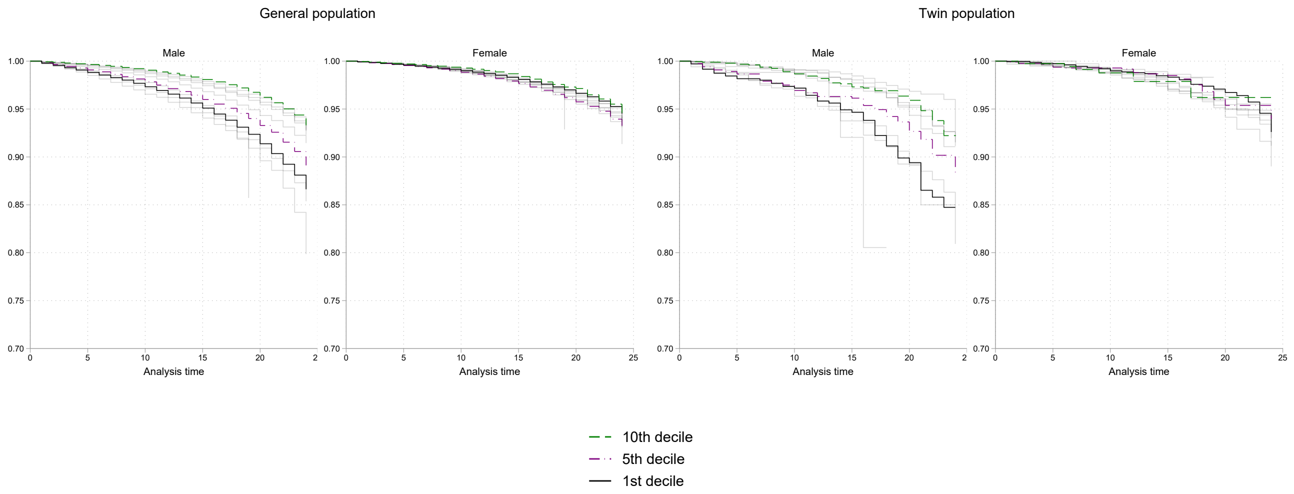

Figure S8: Kaplan-Meier: Survival rates according to earnings and gender

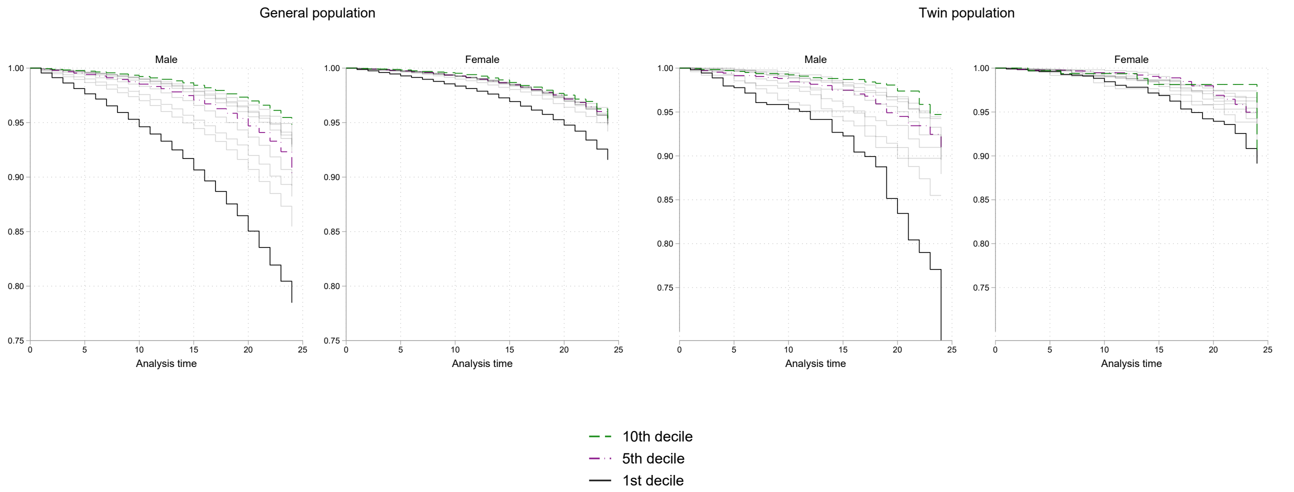

Figure S9: Kaplan-Meier: Survival rates according to education level and gender

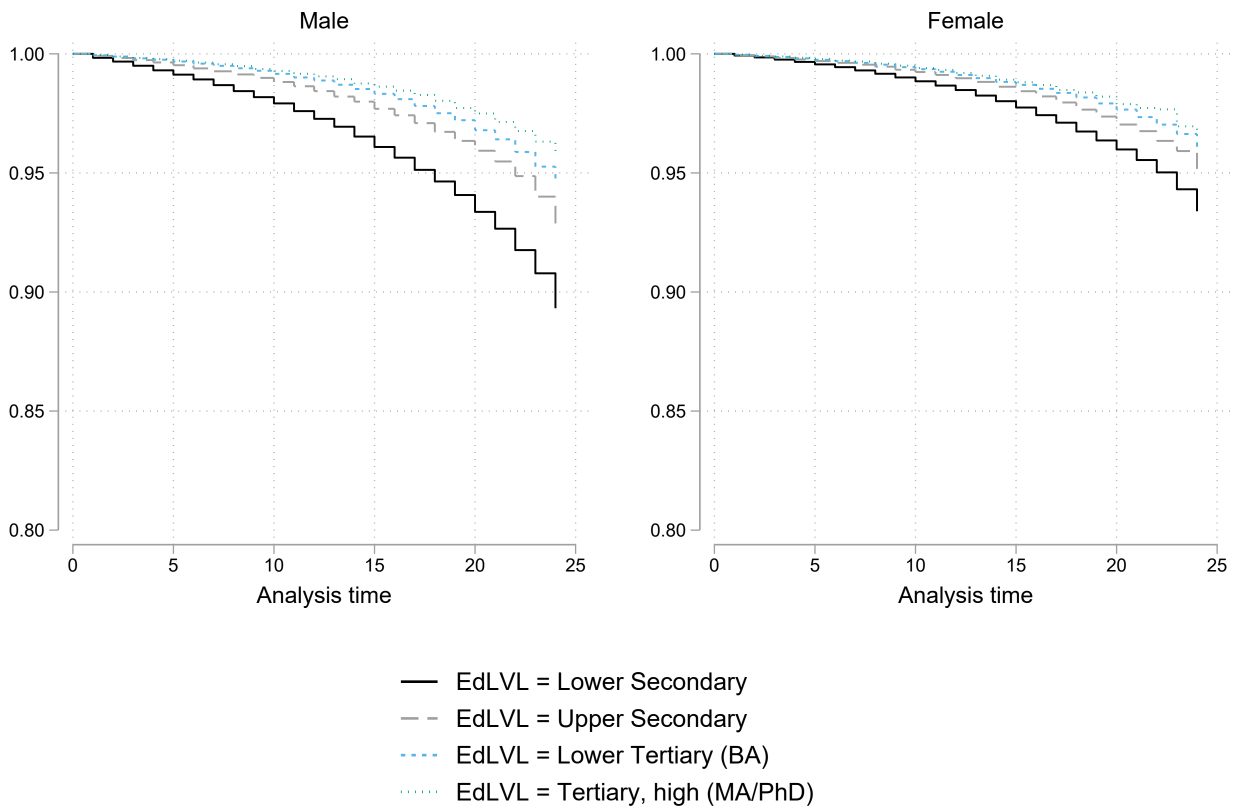

Figure S10: Kaplan-Meier: Survival rates according to immigration background

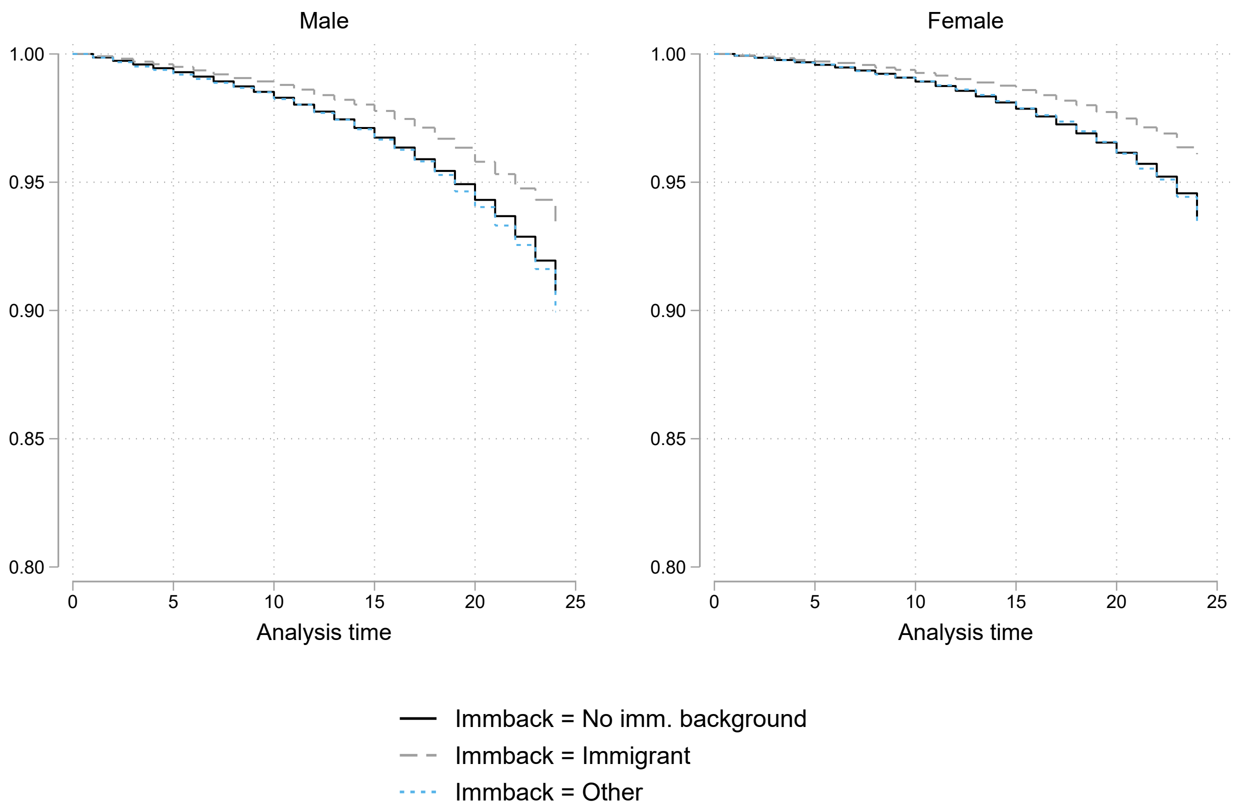

Figure S11: Kaplan-Meier: Survival rates according to birthorder and gender

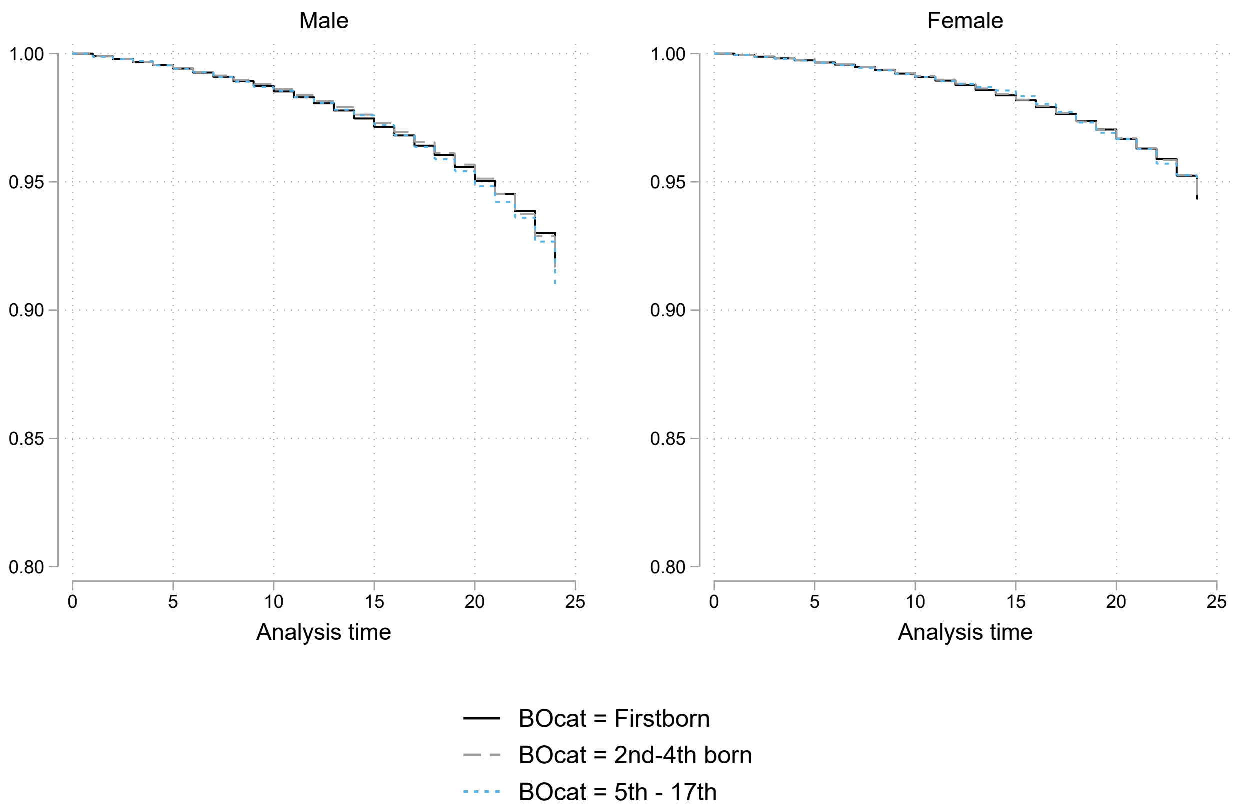

Figure S12: Kaplan-Meier: Survival rates according to number of siblings and gender

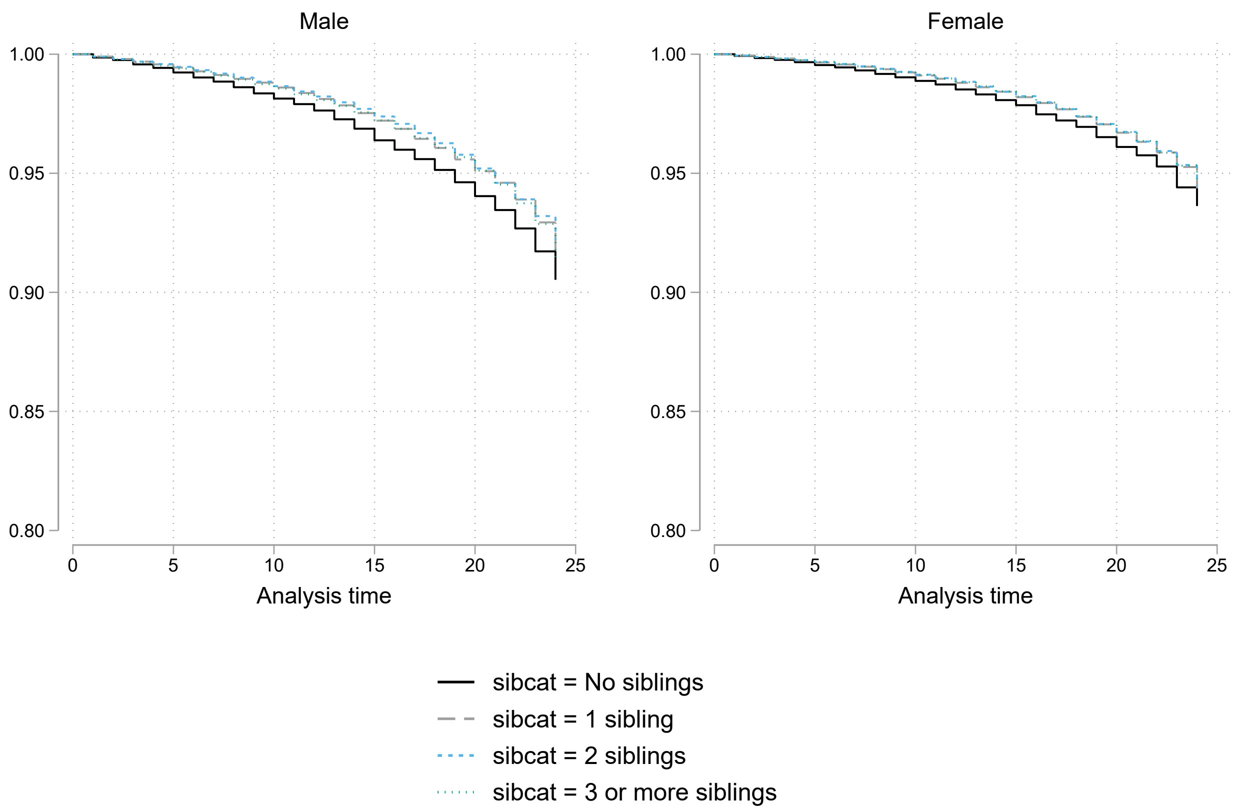

Figure S13: Kaplan-Meier: Survival rates according to net wealth and gender

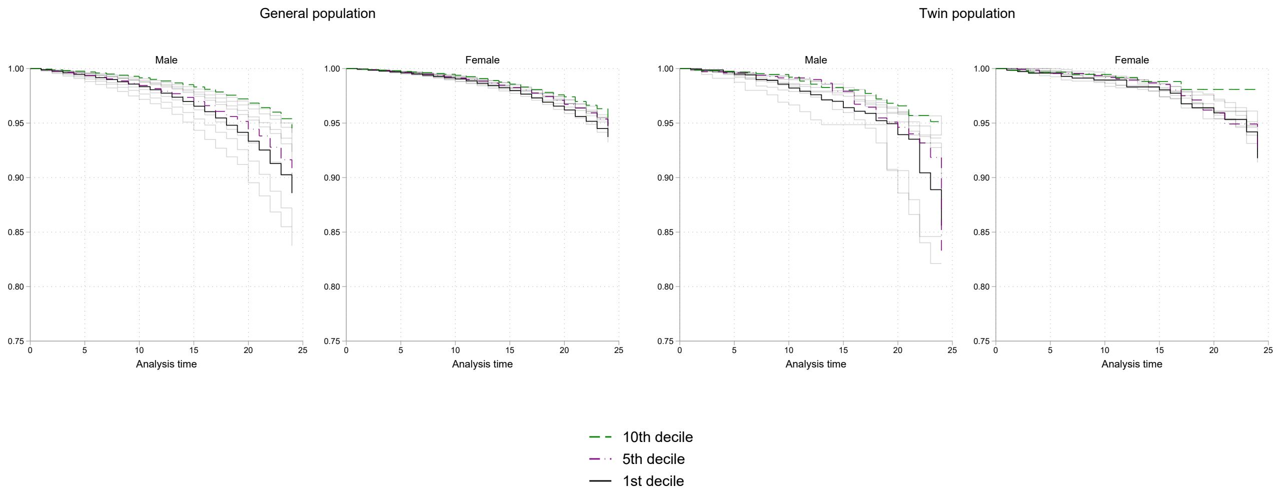

Figure S14: Hazard ratios of mortality according to gross wealth as quintiles. Cox regression models on males and females in the general and twin populations. 95%-Confidence Intervals.

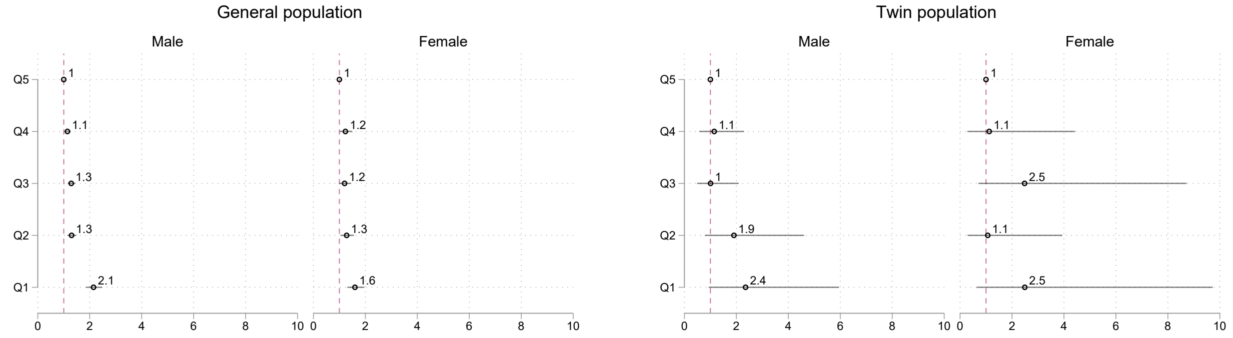

Table S1: Descriptive statistics without listwise deletion: the general population and the population of twins. Immigrants arriving after the age of 38 or nonresidents at the same age are removed. Most missingness are due to lack of family/parental and educational information of immigrants.

	General Population		Twin Population	
	Males	Females	Males	Females
	mean	mean	mean	mean
**Wealth**				
Gross wealth (mill. NOK)	2.247	1.251	2.190	1.162
Net wealth (mill. NOK)	1.183	0.812	1.130	0.734
Debt (mill. NOK)	1.064	0.439	1.060	0.428
Finance capital (mill. NOK)	0.332	0.171	0.285	0.144
Real capital (mill. NOK)	1.915	1.079	1.905	1.018
Gross wealth (cdr)	0.573	0.427	0.590	0.427
Net wealth (rank)	0.516	0.485	0.527	0.484
Missing, wealth	0.011	0.007	0.004	0.003
**Earnings**				
Earnings (100K NOK)	4.949	3.220	4.981	3.235
Earnings (cdr)	0.613	0.378	0.634	0.386
Missing, earnings	0.041	0.073	0.033	0.062
**Immgrant background**				
No imm. background	0.839	0.842	0.934	0.933
Immigrant	0.113	0.108	0.009	0.007
Descendant	0.002	0.002	0.003	0.002
Other immigrant back	0.046	0.047	0.053	0.058
**Family composition**				
Birth order	1.872	1.884	3.308	3.295
Number of siblings	2.163	2.170	2.910	2.920
Missing, family composition	0.103	0.099	0.000	0.000
**Partnership status**				
Nonpartnered	0.507	0.436	0.540	0.467
Partnered	0.480	0.559	0.456	0.530
Missing, partnership status	0.012	0.006	0.003	0.003
**Highest Education**				
Education (yrs)	12.420	12.521	12.318	12.317
Lower Secondary	0.350	0.388	0.380	0.432
Upper Secondary	0.340	0.253	0.351	0.257
Lower Tertiary (BA)	0.190	0.268	0.181	0.256
Tertiary, high (MA/PhD)	0.077	0.054	0.081	0.048
Missing, Education	0.044	0.037	0.008	0.005
Observations	725447	694267	12028	11960

Source: Norwegian adminstrative registers. No missing values on immigrant background.

Table S2: Correlation matrix: the general population

	1.	2.	3.	4.	5.	6.	7.	8.	9.	10.	11.	12.	13.	14.	15.
1.Gross wealth (mill. NOK)	1.00														
2.Net wealth (mill. NOK)	0.98***	1.00													
3.Debt (mill. NOK)	0.34***	0.15***	1.00												
4.Finance capital (mill. NOK)	0.92***	0.94***	0.16***	1.00											
5.Real capital (mill. NOK)	0.60***	0.51***	0.53***	0.24***	1.00										
6.Gross wealth (cdr)	-0.28***	-0.22***	-0.36***	-0.06***	-0.59***	1.00									
7.Net wealth (rank)	0.24***	0.26***	-0.04***	0.05***	0.49***	-0.77***	1.00								
8.Earnings (100K NOK)	0.30***	0.25***	0.36***	0.21***	0.33***	-0.34***	0.15***	1.00							
9.Earnings (cdr)	-0.12***	-0.06***	-0.32***	-0.03***	-0.24***	0.45***	-0.18***	-0.70***	1.00						
10.Education (yrs)	0.08***	0.06***	0.15***	0.02***	0.16***	-0.23***	0.14***	0.31***	-0.35***	1.00					
11.Lower Secondary	-0.07***	-0.05***	-0.15***	-0.02***	-0.14***	0.22***	-0.13***	-0.27***	0.31***	-0.75***	1.00				
12.Upper Secondary	0.02***	0.01***	0.04***	0.00	0.03***	-0.06***	0.04***	0.03***	-0.04***	-0.12***	-0.53***	1.00			
13.Lower Tertiary (BA)	0.03***	0.02***	0.06***	0.01***	0.06***	-0.09***	0.05***	0.13***	-0.16***	0.68***	-0.43***	-0.39***	1.00		
14.Tertiary, high (MA/PhD)	0.06***	0.04***	0.11***	0.02***	0.12***	-0.16***	0.09***	0.23***	-0.25***	0.51***	-0.21***	-0.18***	-0.15***	1.00	
15.Gender	-0.09***	-0.04***	-0.26***	-0.02***	-0.18***	0.29***	-0.07***	-0.29***	0.44***	0.02***	0.03***	-0.10***	0.10***	-0.05***	1.00

* $p < 0.05$, ** $p < 0.01$, *** $p < 0.001$

Table S3: Cox regression models: Gross wealth X Gender interactions tested

	(1) General population		(2) Twin population	
Gross wealth (cdr)	2.96***	[2.64,3.33]	2.09	[0.98,4.48]
Male	1.00	[1.00,1.00]	1.00	[1.00,1.00]
Female	0.75***	[0.68,0.83]	0.95	[0.48,1.91]
Male × Gross wealth (cdr)	1.00	[1.00,1.00]	1.00	[1.00,1.00]
Female × Gross wealth (cdr)	0.42***	[0.36,0.49]	0.40	[0.13,1.20]
Earnings (cdr)	2.51***	[2.29,2.75]	1.93*	[1.05,3.56]
Debt (mill. NOK)	1.07**	[1.02,1.13]	0.97	[0.70,1.33]
Debt (mill. NOK) × Debt (mill. NOK)	0.99*	[0.99,1.00]	1.01	[0.97,1.05]
Lower Secondary	1.00	[1.00,1.00]	1.00	[1.00,1.00]
Upper Secondary	0.79***	[0.75,0.84]	0.86	[0.58,1.27]
Lower Tertiary (BA)	0.73***	[0.67,0.78]	0.88	[0.52,1.49]
Tertiary, high (MA/PhD)	0.70***	[0.61,0.80]	0.66	[0.24,1.82]
Birth order	1.01	[0.99,1.03]	0.99	[0.46,2.10]
Number of siblings	1.06	[0.96,1.16]	1.58	[0.23,10.71]
No imm. background	1.00	[1.00,1.00]	1.00	[1.00,1.00]
Immigrant	0.65	[0.12,3.51]	1.00	[1.00,1.00]
Descendant	0.51	[0.08,3.28]	1.00	[1.00,1.00]
Other	1.10	[0.80,1.51]	1.00	[1.00,1.00]
Observations	1203527		22633	

Exponentiated coefficients; 95% confidence intervals in brackets

* $p < .05$, ** $p < .01$, *** $p < .001$

Table S4: Cox regression models: Gross wealth. Inverted Cumulative Density Ranks (CDR). General population. Robustness checks of different time restrictions according to gender: males observed for 20 years, females for 25 years. Compare with Table 2

	Male (time=20)					Female (time=25)				
	(1)	(2)	(3)	(4)	(5)	(6)	(7)	(8)	(9)	(10)
	Base	Debt	Earnings	Controls	Fam-FE	Base	Debt	Earnings	Controls	Fam-FE
**Financial situation**										
Gross wealth (cdr)	4.71***	3.82***	2.34***	1.97***	2.50***	1.56***	1.88***	1.59***	1.64***	1.74***
	[4.47,4.97]	[3.60,4.05]	[2.20,2.49]	[1.85,2.10]	[2.06,3.04]	[1.45,1.69]	[1.72,2.05]	[1.46,1.74]	[1.50,1.80]	[1.39,2.16]
Debt (mill. NOK)		0.83***	0.90***	0.92***	1.06		1.39***	1.62***	1.22***	1.31**
		[0.81,0.85]	[0.88,0.92]	[0.90,0.94]	[0.99,1.13]		[1.28,1.50]	[1.49,1.75]	[1.13,1.31]	[1.10,1.56]
Debt (mill. NOK) × Debt (mill. NOK)		1.00***	1.00***	1.00***	1.00		0.91***	0.89***	0.97**	0.94*
		[1.00,1.00]	[1.00,1.00]	[1.00,1.00]	[0.99,1.00]		[0.88,0.94]	[0.86,0.92]	[0.94,0.99]	[0.88,1.00]
Earnings (cdr)			3.97***	3.04***	3.05***			2.44***	1.93***	1.95***
			[3.74,4.22]	[2.85,3.24]	[2.55,3.65]			[2.24,2.67]	[1.76,2.12]	[1.58,2.40]
**Highest Education**										
Lower Secondary				1.00	1.00				1.00	1.00
				[1.00,1.00]	[1.00,1.00]				[1.00,1.00]	[1.00,1.00]
Upper Secondary				0.64***	0.77***				0.76***	0.76***
				[0.61,0.66]	[0.69,0.85]				[0.72,0.79]	[0.67,0.86]
Lower Tertiary (BA)				0.59***	0.71***				0.68***	0.72***
				[0.56,0.62]	[0.61,0.83]				[0.64,0.71]	[0.62,0.84]
Tertiary, high (MA/PhD)				0.60***	0.80				0.70***	0.70*
				[0.55,0.65]	[0.62,1.03]				[0.61,0.79]	[0.51,0.97]
**Partnership status**										
Nonpartnered				1.28***	1.35***				1.68***	1.48***
				[1.24,1.33]	[1.23,1.49]				[1.62,1.75]	[1.34,1.63]
Partnered				1.00	1.00				1.00	1.00
				[1.00,1.00]	[1.00,1.00]				[1.00,1.00]	[1.00,1.00]
**Family composition**										
Birth order				0.96***	0.72***				1.00	1.01
				[0.94,0.97]	[0.69,0.74]				[0.98,1.02]	[0.98,1.06]
Number of siblings				0.96***					0.96***	
				[0.95,0.97]					[0.95,0.98]	
**Immigrant background**										
No imm. background				1.00					1.00	
				[1.00,1.00]					[1.00,1.00]	
Immigrant				0.68***					0.95	
				[0.56,0.83]					[0.72,1.25]	
Descendant				1.06					1.12	
				[0.74,1.52]					[0.73,1.72]	
Other				0.99					1.04	
				[0.92,1.07]					[0.95,1.13]	
Observations	513585	513585	513585	513585	513585	583245	583245	583245	583245	583245

Exponentiated coefficients; 95% confidence intervals in brackets

* $p < .05$, ** $p < .01$, *** $p < .001$

Table S5: Cox regression models: Gross wealth X Partnership interactions tested

	(1)		(2)	
	Male		Female	
Gross wealth (cdr)	1.76***	[1.38,2.25]	1.40*	[1.06,1.84]
Nonpartnered	1.19*	[1.04,1.36]	1.12	[0.89,1.41]
Partnered	1.00	[1.00,1.00]	1.00	[1.00,1.00]
Nonpartnered × Gross wealth (cdr)	1.61***	[1.22,2.12]	1.59**	[1.12,2.25]
Partnered × Gross wealth (cdr)	1.00	[1.00,1.00]	1.00	[1.00,1.00]
Earnings (cdr)	2.82***	[2.43,3.28]	1.97***	[1.60,2.43]
Debt (mill. NOK)	1.06	[0.99,1.14]	1.34**	[1.12,1.60]
Debt (mill. NOK) × Debt (mill. NOK)	1.00	[0.99,1.01]	0.94*	[0.88,1.00]
Lower Secondary	1.00	[1.00,1.00]	1.00	[1.00,1.00]
Upper Secondary	0.80***	[0.73,0.87]	0.76***	[0.67,0.86]
Lower Tertiary (BA)	0.71***	[0.62,0.81]	0.72***	[0.62,0.84]
Tertiary, high (MA/PhD)	0.78*	[0.63,0.96]	0.70*	[0.51,0.97]
Birth order	0.99	[0.96,1.02]	1.01	[0.97,1.05]
Number of siblings	0.97	[0.83,1.14]	1.16	[0.94,1.43]
Immigrant	2.26	[0.23,22.47]	0.00***	[0.00,0.00]
Descendant	0.45	[0.03,6.74]	0.00	[0.00,0.00]
Other immigrant background	1.15	[0.67,1.97]	1.19	[0.60,2.34]
Observations	620282		583245	

Exponentiated coefficients; 95% confidence intervals in brackets

* $p < .05$, ** $p < .01$, *** $p < .001$

Table S6: Cox regression models: Partnership status - Gross wealth. Inverted Cumulative Density Ranks (CDR). General population. Underlying Figure 3

	Male with partner				
	(1)	(2)	(3)	(4)	(5)
	Base	Debt	Earnings	Controls	Fam-FE
Gross wealth (cdr)	2.54***	2.61***	2.14***	2.16***	1.86**
	[2.29,2.81]	[2.34,2.90]	[1.92,2.39]	[1.94,2.41]	[1.27,2.72]
Debt (mill. NOK)		1.04	1.09***	1.11***	1.29**
		[1.00,1.08]	[1.04,1.13]	[1.06,1.16]	[1.09,1.54]
Debt (mill. NOK) × Debt (mill. NOK)		1.00	0.99*	0.99**	0.97*
		[0.99,1.00]	[0.99,1.00]	[0.99,1.00]	[0.94,1.00]
Earnings (cdr)			2.40***	1.92***	1.89***
			[2.18,2.65]	[1.73,2.13]	[1.34,2.66]
Lower Secondary				1.00	1.00
				[1.00,1.00]	[1.00,1.00]
Upper Secondary				0.79***	0.73***
				[0.74,0.83]	[0.61,0.87]
Lower Tertiary (BA)				0.63***	0.61***
				[0.58,0.68]	[0.47,0.79]
Tertiary, high (MA/PhD)				0.60***	0.76
				[0.53,0.67]	[0.51,1.15]
Birth order				1.00	1.02
				[0.97,1.02]	[0.95,1.09]
Number of siblings				0.95***	
				[0.93,0.97]	
No imm. background				1.00	
				[1.00,1.00]	
Immigrant				0.86	
				[0.64,1.15]	
Descendant				0.40	
				[0.15,1.07]	
Other				1.10	
				[0.98,1.23]	
Observations	300579	300579	300579	300579	300579

	Female with nopartner				
	(6)	(7)	(8)	(9)	(10)
	Base	Debt	Earnings	Controls	Fam-FE
Gross wealth (cdr)	1.42***	1.54***	1.40***	1.26***	1.18
	[1.27,1.59]	[1.36,1.75]	[1.24,1.59]	[1.11,1.44]	[0.82,1.71]
Debt (mill. NOK)		1.20**	1.32***	1.34***	1.19
		[1.07,1.34]	[1.17,1.48]	[1.19,1.51]	[0.85,1.66]
Debt (mill. NOK) × Debt (mill. NOK)		0.96*	0.94**	0.94**	0.99
		[0.92,1.00]	[0.90,0.98]	[0.90,0.98]	[0.87,1.12]
Earnings (cdr)			1.65***	1.31***	1.79**
			[1.46,1.88]	[1.14,1.50]	[1.23,2.61]
Lower Secondary				1.00	1.00
				[1.00,1.00]	[1.00,1.00]
Upper Secondary				0.76***	0.80*
				[0.71,0.82]	[0.64,0.99]
Lower Tertiary (BA)				0.65***	0.67**
				[0.60,0.70]	[0.51,0.87]
Tertiary, high (MA/PhD)				0.61***	0.54*
				[0.51,0.73]	[0.30,0.97]
Birth order				1.00	0.98
				[0.97,1.02]	[0.92,1.05]
Number of siblings				0.97**	
				[0.95,0.99]	
No imm. background				1.00	
				[1.00,1.00]	
Immigrant				1.01	
				[0.69,1.47]	
Descendant				1.34	
				[0.74,2.43]	
Other				1.08	
				[0.95,1.22]	
Observations	320684	320684	320684	320684	320684

	Male without partner				
	(11)	(12)	(13)	(14)	(15)
	Base	Debt	Earnings	Controls	Fam-FE
Gross wealth (cdr)	5.49***	4.84***	2.89***	2.79***	2.61***
	[5.14,5.86]	[4.51,5.21]	[2.68,3.12]	[2.58,3.00]	[2.00,3.40]
Debt (mill. NOK)		0.88***	0.96**	0.97*	0.98
		[0.86,0.91]	[0.93,0.98]	[0.94,1.00]	[0.89,1.09]
Debt (mill. NOK) × Debt (mill. NOK)		1.00***	1.00	1.00	1.00
		[1.00,1.00]	[1.00,1.00]	[1.00,1.00]	[1.00,1.00]
Earnings (cdr)			3.92***	3.45***	3.34***
			[3.66,4.21]	[3.20,3.71]	[2.65,4.21]
Lower Secondary				1.00	1.00
				[1.00,1.00]	[1.00,1.00]
Upper Secondary				0.76***	0.85*
				[0.73,0.80]	[0.73,0.98]
Lower Tertiary (BA)				0.68***	0.80*
				[0.63,0.72]	[0.64,1.00]
Tertiary, high (MA/PhD)				0.69***	0.72
				[0.62,0.78]	[0.48,1.06]
Birth order				1.01	0.99
				[0.99,1.03]	[0.94,1.04]
Number of siblings				0.96***	
				[0.94,0.97]	
No imm. background				1.00	
				[1.00,1.00]	
Immigrant				0.89	
				[0.69,1.16]	
Descendant				1.45*	
				[1.00,2.11]	
Other				1.02	
				[0.93,1.11]	
Observations	319703	319703	319703	319703	319703

	Female without nopartner				
	(16)	(17)	(18)	(19)	(20)
	Base	Debt	Earnings	Controls	Fam-FE
Gross wealth (cdr)	2.70***	2.70***	2.12***	2.11***	3.05***
	[2.43,3.00]	[2.39,3.06]	[1.87,2.41]	[1.85,2.39]	[1.97,4.71]
Debt (mill. NOK)		1.00	1.17***	1.20***	1.51*
		[0.93,1.09]	[1.07,1.28]	[1.09,1.31]	[1.09,2.09]
Debt (mill. NOK) × Debt (mill. NOK)		1.00	0.98	0.98	0.91
		[0.98,1.02]	[0.95,1.01]	[0.95,1.01]	[0.82,1.01]
Earnings (cdr)			3.24***	2.67***	3.14***
			[2.87,3.65]	[2.35,3.03]	[2.10,4.71]
Lower Secondary				1.00	1.00
				[1.00,1.00]	[1.00,1.00]
Upper Secondary				0.75***	0.67**
				[0.70,0.81]	[0.53,0.86]
Lower Tertiary (BA)				0.70***	0.64**
				[0.65,0.76]	[0.48,0.87]
Tertiary, high (MA/PhD)				0.74**	0.68
				[0.61,0.90]	[0.36,1.27]
Birth order				1.00	0.95
				[0.98,1.03]	[0.88,1.03]
Number of siblings				0.95***	
				[0.93,0.98]	
No imm. background				1.00	
				[1.00,1.00]	
Immigrant				0.86	
				[0.57,1.30]	
Descendant				0.95	
				[0.51,1.76]	
Other				1.00	
				[0.88,1.13]	
Observations	262561	262561	262561	262561	262561

Exponentiated coefficients; 95% confidence intervals in brackets

* $p < 0.05$, ** $p < 0.01$, *** $p < 0.001$

Table S7: Cox regression models: Partnership status - Gross wealth. Inverted Cumulative Density Ranks (CDR). Twin population.

	Male with partner					Female with nopartner				
	(1)	(2)	(3)	(4)	(5)	(6)	(7)	(8)	(9)	(10)
	Base	Debt	Earnings	Controls	Fam-FE	Base	Debt	Earnings	Controls	Fam-FE
Gross wealth (cdr)	3.42***	4.15***	3.78***	3.77***	3.29	1.39	1.71	1.54	1.34	0.27
	[1.68,6.95]	[1.95,8.87]	[1.74,8.20]	[1.73,8.22]	[0.22,49.07]	[0.64,3.02]	[0.72,4.07]	[0.64,3.73]	[0.55,3.26]	[0.01,7.10]
Debt (mill. NOK)		1.28	1.33	1.35	1.35		3.16	3.65*	3.86*	30.32
		[0.88,1.86]	[0.91,1.96]	[0.91,1.98]	[0.32,5.61]		[0.92,10.85]	[1.03,12.98]	[1.08,13.85]	[0.72,1275.28]
Debt (mill. NOK) × Debt (mill. NOK)		0.97	0.97	0.97	0.98		0.51	0.49	0.48	0.19
		[0.91,1.04]	[0.91,1.04]	[0.91,1.04]	[0.71,1.35]		[0.22,1.18]	[0.21,1.14]	[0.20,1.14]	[0.02,1.52]
Earnings (cdr)			1.56	1.26	0.86			1.63	1.03	0.70
			[0.73,3.34]	[0.56,2.86]	[0.06,13.12]			[0.68,3.88]	[0.41,2.56]	[0.04,11.60]
Lower Secondary				1.00	1.00				1.00	1.00
				[1.00,1.00]	[1.00,1.00]				[1.00,1.00]	[1.00,1.00]
Upper Secondary				0.95	0.79				0.72	0.49
				[0.64,1.42]	[0.20,3.08]				[0.44,1.18]	[0.04,5.42]
Lower Tertiary (BA)				1.00	1.06				0.53*	0.10
				[0.60,1.67]	[0.20,5.52]				[0.31,0.89]	[0.00,2.65]
Tertiary, high (MA/PhD)				0.36	0.00				0.16	1.00
				[0.13,1.02]	[0.00,.]				[0.02,1.22]	[1.00,1.00]
Number of siblings				1.02					0.98	
				[0.91,1.14]					[0.86,1.11]	
No imm. background				1.00					1.00	
				[1.00,1.00]					[1.00,1.00]	
Immigrant				1.38					0.00	
				[0.19,9.89]					[0.00,0.00]	
Descendant				0.00					7.83*	
				[0.00,.]					[1.07,57.44]	
Other				0.32					1.21	
				[0.08,1.30]					[0.56,2.59]	
Observations	5413	5413	5413	5413	5413	5999	5999	5999	5999	5999

	Male without partner					Female without nopartner				
	(11)	(12)	(13)	(14)	(15)	(16)	(17)	(18)	(19)	(20)
	Base	Debt	Earnings	Controls	Fam-FE	Base	Debt	Earnings	Controls	Fam-FE
Gross wealth (cdr)	3.69***	3.01***	1.45	1.46	1.07	1.94	2.80*	2.29	2.40	9.16
	[2.30,5.93]	[1.80,5.04]	[0.85,2.47]	[0.86,2.50]	[0.19,6.15]	[0.91,4.13]	[1.16,6.77]	[0.93,5.60]	[0.97,5.91]	[0.17,504.55]
Debt (mill. NOK)		0.78*	0.90	0.90	0.95		1.44	1.67	1.70	1.53
		[0.61,1.00]	[0.71,1.13]	[0.71,1.13]	[0.22,4.14]		[0.86,2.42]	[0.98,2.84]	[0.99,2.93]	[0.01,162.65]
Debt (mill. NOK) × Debt (mill. NOK)		1.01*	1.01	1.01	0.93		0.98	0.97	0.96	3.07
		[1.00,1.02]	[1.00,1.01]	[1.00,1.01]	[0.52,1.65]		[0.88,1.09]	[0.86,1.08]	[0.86,1.08]	[0.08,122.11]
Earnings (cdr)			6.71***	6.00***	3.77			2.84*	2.87*	3.71
			[4.05,11.13]	[3.56,10.11]	[0.69,20.69]			[1.26,6.41]	[1.20,6.86]	[0.08,164.44]
Lower Secondary				1.00	1.00				1.00	1.00
				[1.00,1.00]	[1.00,1.00]				[1.00,1.00]	[1.00,1.00]
Upper Secondary				0.74	1.02				0.59	1.70
				[0.53,1.02]	[0.38,2.72]				[0.35,1.01]	[0.09,30.82]
Lower Tertiary (BA)				0.66	0.40				0.99	0.57
				[0.42,1.06]	[0.07,2.19]				[0.60,1.64]	[0.04,8.49]
Tertiary, high (MA/PhD)				0.90	0.00				1.26	0.82
				[0.41,1.97]	[0.00,.]				[0.43,3.65]	[0.01,95.70]
Number of siblings				0.93					0.96	
				[0.85,1.02]					[0.84,1.09]	
No imm. background				1.00					1.00	
				[1.00,1.00]					[1.00,1.00]	
Immigrant				1.21					0.00	
				[0.17,8.65]					[0.00,0.00]	
Descendant				3.46					0.00	
				[0.85,14.12]					[0.00,0.00]	
Other				0.61					1.16	
				[0.30,1.24]					[0.54,2.50]	
Observations	6092	6092	6092	6092	6092	5129	5129	5129	5129	5129

Exponentiated coefficients; 95% confidence intervals in brackets

* $p < 0.05$, ** $p < 0.01$, *** $p < 0.001$

Table S8: Cox regression models: Net wealth. Inverted Cumulative Density Ranks (CDR). General population.

	Male					Female		
	(1)	(2)	(3)	(4)	(5)	(6)	(7)	(8)
	Base	Income	Controls	Fam-FE	Base	Income	Controls	Fam-FE
Net wealth (rank)	2.81***	2.16***	2.15***	1.81***	1.60***	1.46***	1.38***	1.51***
	[2.68,2.93]	[2.07,2.27]	[2.05,2.25]	[1.59,2.05]	[1.49,1.72]	[1.35,1.57]	[1.28,1.49]	[1.26,1.82]
Earnings (cdr)		5.00***	4.09***	3.78***		2.20***	1.70***	1.84***
		[4.75,5.27]	[3.87,4.32]	[3.29,4.35]		[2.03,2.39]	[1.55,1.85]	[1.51,2.24]
Upper Secondary			0.74***	0.77***			0.74***	0.75***
			[0.72,0.77]	[0.71,0.84]			[0.71,0.78]	[0.67,0.85]
Lower Tertiary (BA)			0.63***	0.68***			0.64***	0.69***
			[0.60,0.66]	[0.60,0.78]			[0.61,0.68]	[0.60,0.80]
Tertiary, high (MA/PhD)			0.62***	0.75**			0.64***	0.64**
			[0.57,0.67]	[0.61,0.92]			[0.56,0.73]	[0.47,0.88]
Birth order			1.00	1.00			1.00	1.05*
			[0.99,1.02]	[0.97,1.04]			[0.98,1.02]	[1.01,1.09]
Number of siblings			0.95***	0.99			0.96***	1.14
			[0.94,0.96]	[0.85,1.16]			[0.95,0.98]	[0.93,1.41]
Number of siblings			1.00				1.00	
			[1.00,1.00]				[1.00,1.00]	
Immigrant			0.80*				0.94	
			[0.66,0.97]				[0.71,1.24]	
Descendant			1.12				1.15	
			[0.79,1.58]				[0.75,1.76]	
Other			1.06				1.06	
			[0.99,1.13]				[0.97,1.16]	
Observations	620282	620282	620282	620282	583245	583245	583245	583245

Exponentiated coefficients; 95% confidence intervals in brackets

* $p < 0.05$, ** $p < 0.01$, *** $p < 0.001$

Table S9: Cox regression models: Net wealth. Inverted Cumulative Density Ranks (CDR). Twin population.

	Male					Female		
	(1)	(2)	(3)	(4)	(5)	(6)	(7)	(8)
	Base	Income	Controls	Twin-FE	Base	Income	Controls	Twin-FE
Net wealth (rank)	2.61***	1.92***	1.92***	1.95	1.41	1.30	1.24	1.37
	[1.89,3.61]	[1.37,2.69]	[1.37,2.70]	[0.90,4.26]	[0.84,2.36]	[0.76,2.20]	[0.72,2.12]	[0.41,4.55]
Earnings (cdr)		5.58***	4.86***	3.27*		1.74*	1.35	1.02
		[3.84,8.09]	[3.27,7.23]	[1.21,8.84]		[1.00,3.03]	[0.74,2.46]	[0.26,3.99]
Upper Secondary			0.80	0.87			0.67*	0.39
			[0.63,1.03]	[0.49,1.57]			[0.47,0.95]	[0.14,1.13]
Lower Tertiary (BA)			0.81	0.70			0.70	0.40
			[0.58,1.14]	[0.30,1.67]			[0.49,1.00]	[0.09,1.78]
Tertiary, high (MA/PhD)			0.58	0.00			0.55	1.19
			[0.31,1.09]	[0.00,.]			[0.22,1.38]	[0.08,18.58]
Number of siblings			0.96				0.97	
			[0.89,1.02]				[0.89,1.06]	
Immigrant			1.09				0.00	
			[0.27,4.39]				[0.00,0.00]	
Descendant			2.19				3.59	
			[0.54,8.82]				[0.50,25.72]	
Other			0.53				1.18	
			[0.28,1.00]				[0.68,2.02]	
Observations	11505	11505	11505	11505	11128	11128	11128	11128

Exponentiated coefficients; 95% confidence intervals in brackets

* $p < 0.05$, ** $p < 0.01$, *** $p < 0.001$

Table S10: Cox regression models: Gross wealth. Quintiles.

	General Population		Twin population	
	(1)	(2)	(3)	(4)
	Male	Female	Male	Female
Q4	1.14*	1.23*	1.15	1.12
	[1.03,1.27]	[1.01,1.50]	[0.58,2.29]	[0.28,4.43]
Q3	1.29***	1.20	1.01	2.48
	[1.15,1.45]	[0.99,1.45]	[0.49,2.09]	[0.71,8.72]
Q2	1.30***	1.28*	1.91	1.07
	[1.14,1.49]	[1.05,1.55]	[0.79,4.61]	[0.29,3.94]
Q1	2.15***	1.60***	2.35	2.49
	[1.86,2.48]	[1.30,1.96]	[0.93,5.95]	[0.64,9.73]
Earnings (cdr)	2.83***	1.97***	2.99*	0.98
	[2.43,3.28]	[1.60,2.43]	[1.06,8.42]	[0.22,4.34]
Debt (mill. NOK)	1.03	1.27**	0.96	1.37
	[0.96,1.11]	[1.07,1.51]	[0.44,2.07]	[0.34,5.61]
Debt (mill. NOK) × Debt (mill. NOK)	1.00	0.94	1.01	1.12
	[0.99,1.01]	[0.89,1.00]	[0.82,1.24]	[0.62,2.03]
Upper Secondary	0.80***	0.76***	0.95	0.41
	[0.73,0.87]	[0.67,0.86]	[0.52,1.75]	[0.14,1.25]
Lower Tertiary (BA)	0.71***	0.72***	0.85	0.46
	[0.63,0.81]	[0.62,0.84]	[0.35,2.07]	[0.10,2.15]
Tertiary, high (MA/PhD)	0.78*	0.71*	0.00	1.47
	[0.63,0.96]	[0.51,0.98]	[0.00,.]	[0.08,27.33]
Birth order	0.99	1.01		
	[0.96,1.03]	[0.97,1.05]		
Number of siblings	0.98	1.16		
	[0.84,1.15]	[0.94,1.43]		
Single	1.43***	1.51***		
	[1.32,1.56]	[1.35,1.69]		
Divorced	1.49***	1.44***		
	[1.34,1.67]	[1.27,1.63]		
Immigrant	2.20	0.00***		
	[0.23,21.52]	[0.00,0.00]		
Descendant	0.49	0.00		
	[0.03,7.27]	[0.00,0.00]		
Other	1.15	1.19		
	[0.67,1.96]	[0.61,2.34]		
Observations	620282	583245	11505	11128

Exponentiated coefficients; 95% confidence intervals in brackets

* $p < 0.05$, ** $p < 0.01$, *** $p < 0.001$

Table S11: Cox regression models: Finance and real capital. Inverted Cumulative Density Ranks (CDR). General population.

	Male				Female			
	(1)	(2)	(3)	(4)	(5)	(6)	(7)	(8)
	Wealth	Debt	Earnings	Full	Wealth	Debt	Earnings	Full
Finance capital	2.44***	2.47***	1.80***	1.68***	1.78***	1.77***	1.50***	1.57***
	[2.30,2.58]	[2.33,2.61]	[1.70,1.91]	[1.44,1.95]	[1.66,1.92]	[1.65,1.90]	[1.39,1.62]	[1.30,1.89]
Real capital	3.57***	3.16***	1.92***	1.88***	1.16***	1.36***	1.35***	1.48***
	[3.36,3.79]	[2.96,3.37]	[1.79,2.05]	[1.58,2.24]	[1.08,1.26]	[1.24,1.49]	[1.23,1.48]	[1.20,1.84]
Debt (mill. NOK)		0.90***	1.00	1.04		1.31***	1.16***	1.28**
		[0.88,0.92]	[0.97,1.02]	[0.98,1.10]		[1.21,1.42]	[1.07,1.25]	[1.07,1.52]
Debt (mill. NOK) × Debt (mill. NOK)		1.00***	1.00	1.00		0.93***	0.98*	0.94
		[1.00,1.00]	[1.00,1.00]	[0.99,1.00]		[0.90,0.96]	[0.95,1.00]	[0.89,1.00]
Earnings (cdr)			2.81***	2.77***			1.85***	1.87***
			[2.65,2.98]	[2.38,3.21]			[1.68,2.03]	[1.51,2.31]
Upper Secondary			0.79***	0.80***			0.77***	0.77***
			[0.76,0.82]	[0.73,0.88]			[0.73,0.81]	[0.68,0.87]
Lower Tertiary (BA)			0.69***	0.72***			0.70***	0.73***
			[0.66,0.73]	[0.63,0.82]			[0.66,0.74]	[0.63,0.85]
Tertiary, high (MA/PhD)			0.71***	0.82			0.73***	0.72*
			[0.66,0.77]	[0.66,1.01]			[0.64,0.83]	[0.52,0.99]
Birth order			1.00	0.99			1.00	1.02
			[0.99,1.02]	[0.96,1.02]			[0.98,1.02]	[0.98,1.06]
Number of siblings			0.95***	0.97			0.96***	1.16
			[0.94,0.96]	[0.83,1.14]			[0.94,0.97]	[0.94,1.43]
Nonpartnered			1.47***	1.45***			1.70***	1.50***
			[1.42,1.52]	[1.34,1.56]			[1.64,1.77]	[1.37,1.65]
Number of siblings			1.00					
			[1.00,1.00]					
Immigrant			0.83				0.94	
			[0.69,1.02]				[0.71,1.24]	
Descendant			1.09				1.12	
			[0.77,1.55]				[0.73,1.71]	
Other			1.05				1.04	
			[0.98,1.12]				[0.95,1.13]	
Number of siblings							1.00	
							[1.00,1.00]	
Observations	620282	620282	620282	620282	583245	583245	583245	583245

Exponentiated coefficients; 95% confidence intervals in brackets

* $p < 0.05$, ** $p < 0.01$, *** $p < 0.001$

Table S12: Cox regression models: Finance and real capital. Inverted Cumulative Density Ranks (CDR). Twin population.

	Male				Female			
	(1)	(2)	(3)	(4)	(5)	(6)	(7)	(8)
	Wealth	Debt	Earnings	Full	Wealth	Debt	Earnings	Full
Finance capital	2.20***	2.25***	1.65*	2.84	2.57***	2.56***	2.28**	3.06
	[1.48,3.29]	[1.51,3.37]	[1.09,2.48]	[0.89,9.12]	[1.55,4.26]	[1.54,4.25]	[1.34,3.88]	[0.82,11.43]
Real capital	3.09***	2.73***	1.50	1.73	0.85	1.15	1.18	1.90
	[2.02,4.74]	[1.71,4.34]	[0.92,2.46]	[0.55,5.45]	[0.50,1.45]	[0.61,2.15]	[0.62,2.26]	[0.46,7.81]
Debt (mill. NOK)		0.89	1.00	0.81		1.57	1.46	1.57
		[0.75,1.05]	[0.85,1.18]	[0.37,1.77]		[0.91,2.71]	[0.85,2.50]	[0.39,6.37]
Debt (mill. NOK) × Debt (mill. NOK)		1.01	1.00	1.05		0.90	0.94	1.03
		[1.00,1.01]	[1.00,1.01]	[0.86,1.29]		[0.72,1.13]	[0.78,1.14]	[0.57,1.84]
Earnings (cdr)			3.67***	2.82			1.50	1.25
			[2.39,5.64]	[0.97,8.23]			[0.79,2.85]	[0.28,5.51]
Upper Secondary			0.84	0.94			0.69*	0.42
			[0.66,1.08]	[0.51,1.73]			[0.48,0.99]	[0.14,1.22]
Lower Tertiary (BA)			0.87	0.78			0.78	0.43
			[0.62,1.22]	[0.33,1.87]			[0.54,1.12]	[0.09,2.01]
Tertiary, high (MA/PhD)			0.65	0.00			0.65	1.02
			[0.35,1.22]	[0.00,.]			[0.25,1.65]	[0.06,17.74]
Birth order			0.99	0.00			1.14	231029.32
			[0.88,1.11]	[0.00,0.00]			[0.98,1.34]	[231029.32,231029.32]
Nonpartnered			1.48**	1.22			1.49**	1.12
			[1.17,1.86]	[0.70,2.12]			[1.13,1.95]	[0.60,2.08]
Number of siblings			0.97				0.88	
			[0.88,1.07]				[0.77,1.01]	
Immigrant			1.18				0.00	
			[0.29,4.79]				[0.00,.]	
Descendant			2.10				3.74	
			[0.52,8.48]				[0.52,26.81]	
Other			0.53*				1.18	
			[0.28,0.99]				[0.69,2.03]	
Observations	11505	11505	11505	11505	11128	11128	11128	11128

Exponentiated coefficients; 95% confidence intervals in brackets

* $p < 0.05$, ** $p < 0.01$, *** $p < 0.001$

Table S13: Cox regression models: Gross wealth. Inverted Cumulative Density Ranks (CDR). General population. Robustness checks of different time restrictions:Time in 5,10,15. Compare with Table 2

	Male			Female		
	t=5	t=10	t=15	t=5	t=10	t=15
Gross wealth (cdr)	0.865	1.659*	1.611***	1.758	2.422**	1.907***
	(-0.18)	(2.19)	(3.75)	(0.51)	(2.91)	(3.72)
Earnings (cdr)	1.204	1.960**	2.622***	10.18	1.710	1.852***
	(0.28)	(3.05)	(7.82)	(1.89)	(1.72)	(3.59)
Debt (mill. NOK)	0.434**	0.744***	0.835***	2.531	1.525	1.227
	(-2.75)	(-5.02)	(-4.95)	(0.87)	(1.63)	(1.44)
Debt (mill. NOK) × Debt (mill. NOK)	1.066	1.001***	1.001**	0.518	0.790*	0.905
	(1.83)	(3.63)	(2.66)	(-1.35)	(-2.34)	(-1.79)
Lower Secondary	1	1	1	1	1	1
	(.)	(.)	(.)	(.)	(.)	(.)
Upper Secondary	0.341*	0.616***	0.580***	0.406	0.484***	0.513***
	(-2.31)	(-3.74)	(-7.61)	(-1.50)	(-4.28)	(-6.82)
Lower Tertiary (BA)	0.205*	0.414***	0.529***	0.106*	0.377***	0.463***
	(-2.40)	(-4.47)	(-5.81)	(-2.18)	(-4.24)	(-6.00)
Tertiary, high (MA/PhD)	0.0919*	0.431**	0.523***	0.0802	0.267**	0.295***
	(-2.23)	(-2.71)	(-3.56)	(-1.59)	(-3.18)	(-4.72)
Observations	59025	216826	373529	56050	206255	353024

Exponentiated coefficients; t statistics in parentheses

* $p < .05$, ** $p < .01$, *** $p < .001$

Table S14: Cox regression models: Gross wealth. Inverted Cumulative Density Ranks (CDR). General population. Models controlled for high density cities

	Male					Female				
	(1)	(2)	(3)	(4)	(5)	(6)	(7)	(8)	(9)	(10)
	Base	Debt	Income	Controls	Fam-FE	Base	Debt	Income	Controls	Fam-FE
Gross wealth (cdr)	5.76***	5.30***	3.26***	3.12***	2.76***	1.56***	1.88***	1.59***	1.53***	1.66***
	[5.47,6.06]	[5.01,5.60]	[3.07,3.46]	[2.94,3.30]	[2.35,3.24]	[1.45,1.69]	[1.72,2.05]	[1.46,1.74]	[1.40,1.68]	[1.33,2.06]
Debt (mill. NOK)		0.93***	0.99	1.00	1.03		1.39***	1.62***	1.58***	1.54***
		[0.91,0.95]	[0.97,1.01]	[0.98,1.02]	[0.97,1.10]		[1.28,1.50]	[1.49,1.75]	[1.46,1.71]	[1.28,1.86]
Debt (mill. NOK) × Debt (mill. NOK)		1.00***	1.00	1.00	1.00		0.91***	0.89***	0.90***	0.90**
		[1.00,1.00]	[1.00,1.00]	[1.00,1.00]	[0.99,1.00]		[0.88,0.94]	[0.86,0.92]	[0.87,0.93]	[0.83,0.97]
Earnings (cdr)			3.68***	3.15***	3.03***			2.44***	2.03***	2.06***
			[3.48,3.89]	[2.97,3.34]	[2.62,3.52]			[2.24,2.67]	[1.85,2.22]	[1.67,2.53]
Upper Secondary				0.76***	0.78***				0.73***	0.76***
				[0.74,0.79]	[0.72,0.85]				[0.69,0.76]	[0.67,0.85]
Lower Tertiary (BA)				0.64***	0.69***				0.62***	0.69***
				[0.61,0.67]	[0.61,0.79]				[0.59,0.65]	[0.60,0.80]
Tertiary, high (MA/PhD)				0.63***	0.77*				0.59***	0.62**
				[0.58,0.68]	[0.62,0.95]				[0.52,0.67]	[0.45,0.86]
Birth order				1.00					1.00	
				[0.99,1.02]					[0.98,1.02]	
Number of siblings				0.95***					0.97***	
				[0.94,0.97]					[0.95,0.98]	
Immigrant				0.72**					0.85	
				[0.59,0.88]					[0.64,1.12]	
Other				1.03					1.03	
				[0.96,1.10]					[0.94,1.12]	
Oslo				1.24***	1.25**				1.46***	1.31**
				[1.18,1.29]	[1.08,1.44]				[1.37,1.54]	[1.10,1.55]
Bergen				1.02	0.94				1.09*	1.14
				[0.95,1.09]	[0.74,1.20]				[1.00,1.19]	[0.86,1.53]
Stavanger				1.24***	1.01				1.22***	1.52*
				[1.13,1.37]	[0.74,1.36]				[1.09,1.37]	[1.04,2.21]
Trondheim				0.85***	0.79				1.14*	1.51**
				[0.78,0.93]	[0.59,1.05]				[1.03,1.25]	[1.11,2.06]
Observations	620282	620282	620282	620282	620282	583245	583245	583245	583245	583245

Exponentiated coefficients; 95% confidence intervals in brackets

* $p < 0.05$, ** $p < 0.01$, *** $p < 0.001$

Table S15: Cox regression models: Gross wealth. Inverted Cumulative Density Ranks (CDR). Twin population. Models controlled for high density cities

	Male					Female				
	(1)	(2)	(3)	(4)	(5)	(6)	(7)	(8)	(9)	(10)
	Base	Debt	Income	Controls	Twin-FE	Base	Debt	Income	Controls	Twin-FE
Gross wealth (cdr)	4.81***	4.46***	2.54***	2.35***	3.07*	1.29	1.81	1.55	1.47	2.93
	[3.32,6.96]	[2.99,6.66]	[1.67,3.87]	[1.54,3.58]	[1.04,9.10]	[0.76,2.18]	[0.98,3.33]	[0.83,2.89]	[0.78,2.76]	[0.63,13.60]
Debt (mill. NOK)		0.92	1.01	1.01	0.97		1.70	1.95*	1.94*	2.08
		[0.78,1.08]	[0.86,1.18]	[0.87,1.18]	[0.46,2.07]		[0.98,2.94]	[1.10,3.44]	[1.11,3.41]	[0.53,8.17]
Debt (mill. NOK) × Debt (mill. NOK)		1.00	1.00	1.00	1.00		0.88	0.86	0.87	0.96
		[1.00,1.01]	[1.00,1.01]	[1.00,1.01]	[0.82,1.23]		[0.70,1.11]	[0.67,1.09]	[0.69,1.09]	[0.55,1.65]
Earnings (cdr)			4.48***	4.03***	2.66			2.13*	1.72	1.09
			[2.98,6.75]	[2.64,6.17]	[0.94,7.51]			[1.17,3.85]	[0.91,3.26]	[0.25,4.65]
Upper Secondary				0.81	0.92				0.65*	0.38
				[0.64,1.04]	[0.50,1.69]				[0.46,0.93]	[0.13,1.12]
Lower Tertiary (BA)				0.79	0.75				0.70*	0.26
				[0.56,1.11]	[0.31,1.84]				[0.49,0.99]	[0.05,1.32]
Tertiary, high (MA/PhD)				0.55	0.00				0.54	0.76
				[0.30,1.04]	[0.00,.]				[0.21,1.36]	[0.04,13.29]
Immigrant				0.88					0.00	
				[0.22,3.58]					[0.00,.]	
Other				0.58					1.22	
				[0.33,1.03]					[0.73,2.07]	
Oslo				1.64**	1.11				1.20	0.63
				[1.21,2.23]	[0.37,3.36]				[0.81,1.78]	[0.20,1.94]
Bergen				1.12	0.46				1.04	3.34
				[0.73,1.72]	[0.08,2.52]				[0.58,1.88]	[0.29,38.71]
Stavanger				1.47	0.55				1.08	1.68
				[0.80,2.70]	[0.04,6.82]				[0.48,2.44]	[0.14,19.66]
Trondheim				0.76	1.66				0.92	2.75
				[0.40,1.44]	[0.10,26.61]				[0.45,1.86]	[0.12,63.13]
Observations	11505	11505	11505	11505	11505	11128	11128	11128	11128	11128

Exponentiated coefficients; 95% confidence intervals in brackets

* $p < 0.05$, ** $p < 0.01$, *** $p < 0.001$

Table S16: Cox regression models: Gross wealth. Inverted Cumulative Density Ranks (CDR). General population. Controlled for 1 and 4 digits occupational ISCO-88 codes

	Male			Female		
	(1)	(2)	(3)	(4)	(5)	(6)
	IncDebtDem	ISCO1FE	ISCO4FE	IncDebtDem	ISCO1FE	ISCO4FE
Gross wealth (cdr)	2.52***	2.49***	2.44***	1.12	1.10	1.09
	[2.27,2.80]	[2.24,2.77]	[2.19,2.71]	[0.97,1.29]	[0.95,1.27]	[0.94,1.26]
Earnings (cdr)	1.58***	1.53***	1.54***	0.89	0.83*	0.81**
	[1.42,1.77]	[1.36,1.71]	[1.37,1.73]	[0.77,1.03]	[0.71,0.96]	[0.70,0.95]
Debt (mill. NOK)	1.09***	1.10***	1.10***	1.46***	1.46***	1.45***
	[1.04,1.15]	[1.05,1.16]	[1.04,1.16]	[1.26,1.69]	[1.27,1.69]	[1.26,1.68]
Debt (mill. NOK) × Debt (mill. NOK)	0.99	0.99	0.99	0.90**	0.90**	0.90**
	[0.99,1.00]	[0.99,1.00]	[0.99,1.00]	[0.84,0.97]	[0.84,0.97]	[0.84,0.97]
Upper Secondary	0.80***	0.83***	0.85***	0.78***	0.81***	0.84***
	[0.76,0.85]	[0.78,0.88]	[0.80,0.90]	[0.72,0.84]	[0.75,0.88]	[0.77,0.91]
Lower Tertiary (BA)	0.70***	0.73***	0.77***	0.66***	0.70***	0.71***
	[0.65,0.75]	[0.68,0.79]	[0.71,0.84]	[0.61,0.71]	[0.64,0.76]	[0.65,0.78]
Tertiary, high (MA/PhD)	0.58***	0.61***	0.67***	0.56***	0.58***	0.59***
	[0.51,0.65]	[0.54,0.70]	[0.58,0.76]	[0.46,0.69]	[0.47,0.71]	[0.47,0.74]
Birth order	1.00	1.00	1.00	1.00	1.00	1.00
	[0.97,1.02]	[0.97,1.02]	[0.98,1.03]	[0.97,1.03]	[0.97,1.03]	[0.97,1.03]
Number of siblings	0.95***	0.95***	0.95***	0.97*	0.97*	0.97*
	[0.93,0.97]	[0.93,0.97]	[0.93,0.97]	[0.95,1.00]	[0.95,1.00]	[0.95,0.99]
Immigrant	1.17	1.15	1.12	1.36	1.33	1.31
	[0.81,1.67]	[0.81,1.65]	[0.78,1.60]	[0.85,2.19]	[0.83,2.15]	[0.81,2.11]
Other	1.05	1.06	1.05	1.04	1.05	1.04
	[0.94,1.18]	[0.94,1.18]	[0.94,1.18]	[0.91,1.20]	[0.91,1.20]	[0.91,1.20]
Observations	383817	383817	383817	369351	369351	369351

Exponentiated coefficients; 95% confidence intervals in brackets

* $p < 0.05$, ** $p < 0.01$, *** $p < 0.001$
